# Molecular Mechanisms Underlying Freezing Tolerance in Plants: Implications for Cryopreservation

**DOI:** 10.3390/ijms251810110

**Published:** 2024-09-20

**Authors:** Magdalena Białoskórska, Anna Rucińska, Maja Boczkowska

**Affiliations:** 1 Plant Breeding and Acclimatization Institute—National Research Institute in Radzików, 05-870 Błonie, Poland; m.bialoskorska@ihar.edu.pl (M.B.); a.rucinska@ihar.edu.pl (A.R.); 2 Botanical Garden, Center for Biological Diversity Conservation in Powsin, Polish Academy of Science, Prawdziwka 2, 02-976 Warszawa, Poland

**Keywords:** cryopreservation, freezing tolerance, molecular mechanisms, cold acclimatization, *COR*, CBFs, ICE, ncRNA, antifreeze proteins

## Abstract

Cryopreservation is a crucial technique for the long-term ex situ conservation of plant genetic resources, particularly in the context of global biodiversity decline. This process entails freezing biological material at ultra-low temperatures using liquid nitrogen, which effectively halts metabolic activities and preserves plant tissues over extended periods. Over the past seven decades, a plethora of techniques for cryopreserving plant materials have been developed. These include slow freezing, vitrification, encapsulation dehydration, encapsulation–vitrification, droplet vitrification, cryo-plates, and cryo-mesh techniques. A key challenge in the advancement of cryopreservation lies in our ability to understand the molecular processes underlying plant freezing tolerance. These mechanisms include cold acclimatization, the activation of cold-responsive genes through pathways such as the *ICE–CBF–COR* cascade, and the protective roles of transcription factors, non-coding RNAs, and epigenetic modifications. Furthermore, specialized proteins, such as antifreeze proteins (AFPs) and late embryogenesis abundant (LEA) proteins, play crucial roles in protecting plant cells during freezing and thawing. Despite its potential, cryopreservation faces significant challenges, particularly in standardizing protocols for a wide range of plant species, especially those from tropical and subtropical regions. This review highlights the importance of ongoing research and the integration of omics technologies to improve cryopreservation techniques, ensuring their effectiveness across diverse plant species and contributing to global efforts regarding biodiversity conservation.

## 1. Introduction

The diversity of life on Earth, both in terrestrial and aquatic ecosystems, is declining at an alarming rate [1,2]. The current extinction event is the sixth in the last 550 million years and may be the most severe in the planet’s history. It may exceed the scale of the previous five major extinctions in regards to the number of species affected [3,4]. Natural populations are exposed to many threats, including those arising from climate change, pollution, the emergence of novel pathogens, pests, invasive species, habitat loss, and over-harvesting [5,6,7]. Two principal strategies have been developed to address these challenges and ensure biodiversity preservation, i.e., *in situ* and *ex situ*. The former aims to protect species within their natural habitats, while the latter is focused on preserving species outside their natural habitats. Plant *ex situ* conservation includes preserving plant specimens in several collections, such as botanical gardens, germplasm banks (which preserve seeds, pollen, tissues, and organs), and field collections of vegetatively propagated crop species [8,9,10].

Cryopreservation is one of the most recent techniques for ex situ biodiversity conservation. The term “cryopreservation” describes a process whereby biological material is frozen and stored at extremely low temperatures [11,12]. This method employs liquid nitrogen (LN), which enables the maintenance of plant material at exceedingly low temperatures, specifically at −196 °C in liquid and at −150 °C in vapor over liquid. The utilization of LN presents many benefits, including relatively low cost, chemical inertness, accessibility, and a high degree of independence from electricity [11]. In the context of cryogenic conditions, the reduction or cessation of kinetic energy and molecular motion in biological systems has the effect of weakening transport and enzymatic reactions, which in turn results in the slowing of the aging process [13]. Nevertheless, the cryopreservation protocol must be optimized case-by-case, depending on the species and type of explant to be frozen. This is due to the necessity of implementing an effective method to prevent ice crystals from forming from the water in the cells. The formation and growth of ice crystals intracellularly cause cell membrane rupture, leading to loss of function and ultimately, to cell death [14]. Advances in mammalian systems in recent years have influenced the development of cryopreservation strategies for plant cells and organs [15]. 

Cryopreservation protocols have been developed for many species, primarily those of economic importance [16,17,18,19]. Nevertheless, disparities in tolerance to cryopreservation have been identified, as evidenced by the differential capacity for post-cryopreservation regeneration [20,21,22]. These observations suggest the existence of molecular-level mechanisms underlying freezing tolerance and metabolic resuscitation after thawing. This paper provides a comprehensive overview of this field’s current state of knowledge.

## 2. Overview of Cryopreservation Techniques

Since the initial paper on the cryopreservation of plant material was published in 1956, many techniques and protocols have been developed, offering an array of methods for effectively storing plant germplasm [23]. 

### 2.1. Slow Freezing

One of the most traditional cryopreservation techniques is slow freezing, also called controlled-rate freezing. This method has been extensively employed for the conservation of plant genetic resources. This method gradually cools the plant material at a rate of no more than 2 °C/min until the temperature reaches −40 °C, after which it is immersed in LN [18]. Consequently, at the outset of the process, the cells are subcooled instead of frozen. The cytoplasm remains unfrozen due to the concentration of solutes, and the cell wall protects the cell membrane from damaging ice crystals [24]. As the temperature decreases, the proportion of the extracellular solution that undergoes ice formation increases, leading to an elevation in the osmotic pressure gradient. This is counterbalanced by water migration from the cells into the intercellular spaces, where it freezes. Under optimal conditions, most of the water from the cells is removed, preventing the formation of intracellular ice crystals following subsequent immersion in LN [25]. Several controlled-rate freezing techniques have been developed to date [26]. This technique is effective for winter dormant buds, shoot tips from temperate and subtropical plants, undifferentiated cell suspensions, and calluses [15,27,28,29]. Modifying this method by pre-treatment of the plant material with cryoprotectant solutions allows its application to a broader range of species and improves regeneration efficiency after cryopreservation [13,18,30]. The utilization of standard operating procedures, programmed cooling rates, large-scale batch processing, and efficient use of working time are among the key benefits of this method [28]. However, there are significant drawbacks. Freezing rates require precise control through sophisticated and costly programmable freezers, and the concentration of cryoprotectant agents must also be carefully regulated [31]. Additionally, some cryoprotectant agents can be toxic to specific plant tissues, necessitating the development of protocols tailored to the species and tissues [32]. 

#### Cryopreservation of Dormant Buds

Cryopreservation of dormant buds is a protocol based on slow freezing. Its history dates back to the 1960s [33]. Since then, the protocol has been continuously modified and improved [27,34,35,36]. Its uniqueness lies in the fact that this method can be applied to preserving trees and shrubs native to temperate climates that undergo natural dormancy during winter [16]. Exposure to photoperiod and/or low temperatures induces this dormant phase, and plants must demonstrate moderate to high winter hardiness to be eligible for cryopreservation [37]. The protocol takes advantage of the natural acclimation of buds on the parent plant and the physical dehydration and slow freezing of the nodal section of the branch. The buds are collected and subjected to artificial acclimatization to cold conditions in the winter months. They are then dried in air, placed in cryovials, and exposed to slow freezing. Following thawing and rehydration of the buds, they are directly grafted onto rootstocks [38]. The absence of an *in vitro* culture stage in the protocol results in a notable reduction in the overall length of the process, a diminished risk of infection, and a decrease in associated costs [37]. In addition, this method does not utilize cryoprotectants and allows for the preservation of large amounts of material with minimal effort. However, it requires a programmable freezer that precisely controls the initial phase of slow freezing. Another problem is that genotypes within a species have different levels of freeze tolerance, resulting in different success rates [36,39].

### 2.2. Vitrification

Another cryopreservation method is vitrification. This methodology was developed and first implemented in the 1990s [40,41]. In general, vitrification can be defined as the kinetic transformation of a substance from a liquid to a glassy state [42]. During cryopreservation, biological materials are treated with a combination of highly concentrated penetrating and nonpenetrating cryoprotectants at 0 °C. This is followed by immersion in LN, which facilitates the rapid cooling process. During this phase, the viscous cryoprotectant solidifies into metastable glass, avoiding the formation of crystals [28]. Cryoprotectants enhance the viscosity of cells, thereby inhibiting the formation of ice crystals both within and outside the cell. This reduces the risk of damage to cellular organelle structures, as ice formation is prevented [28]. Cell membranes are protected by a gelatinous fluid that forms a glassy state, which enables cells to survive at cryogenic temperatures [43]. Furthermore, vitrification solutions assist in preventing additional lethal water loss and enhance the regeneration percentage after cryopreservation [44]. The high viscosity of this glass hinders the progression of chemical reactions that rely on molecular diffusion. Consequently, its formation impedes metabolic activity, leading to its stability over time [45]. One of the most notable benefits of vitrification is its extensive applicability to a diverse range of plant species and tissue types, including shoot tips and somatic and zygotic embryos [46,47,48]. Moreover, the technique is straightforward and necessitates minimal expenditure on costly equipment, conferring notable benefits in regards to cost-effectiveness and feasibility. The most substantial drawback is the toxicity of cryoprotectants, as previously discussed.

### 2.3. Encapsulation Dehydration

Simultaneously, encapsulation dehydration technology was developed in the 1990s [49]. It combines synthetic seed production and dehydration technologies [50]. Explants are encapsulated in gel beads, usually calcium alginate, placed in a high-sucrose liquid medium for osmotic dehydration, partially dried over silica gel or in a laminar flow chamber for physical dehydration, and placed in LN [51]. Depending on the species, desiccated capsules are either directly immersed in LN or subjected to a slow-freezing process [52]. Enclosing the explants in capsules allows them to survive dehydration. Most of the frozen water is removed from the cells during this process. During immersion in LN, vitrification of the dissolved internal substances occurs, preventing ice crystallization and resulting in a high percentage of regeneration after thawing [53]. This method is widely applicable. It can be adapted to different species and explant types. It exhibits low toxicity compared to that of other methods. Encapsulation protects explants from mechanical damage by providing an additional layer. However, the encapsulation process requires precise control of the degree of dehydration. It must be optimized and is labor- and time-consuming, mainly when used for extensive germplasm collections [51].

### 2.4. Encapsulation–Vitrification

The increased interest in using cryopreservation methodologies has resulted in continuous development of new techniques. One such technique is encapsulation–vitrification, which combines the encapsulation dehydration and vitrification protocols. This procedure was developed by Tannoury et al. in 1991 [54]. The encapsulation step protects explants during the procedure and reduces the harmful effects of both osmotic stress and cryoprotectant toxicity. A significant advantage of this method is that it allows cryopreservation through direct immersion in LN, eliminating the need to purchase programmable freezers [55]. This method also applies to explants from tropical or subtropical species, with which other cryopreservation methods often prove ineffective [16,56,57,58].

### 2.5. Droplet Vitrification

In 2000, Pennycooke and Towill published a subsequent cryopreservation protocol designated as droplet vitrification [59]. The method was based on the technique described by Kartha et al. for the cryopreservation of apical meristems in droplets of dimethyl sulfoxide (DMSO) [60]. It involves the placement of small explants, such as shoot tips or embryos, in a droplet of vitrification solution on a strip of aluminum foil, followed by incubation in the cryoprotectant mixture and rapid cooling by imbibition of liquid nitrogen [59]. The solution includes DMSO, ethylene glycol, glycerol, and MS medium with sucrose, placed as a droplet on the aluminum foil surface [61]. Rapid cooling and heating rates are achieved, reaching up to 130 °C per second, facilitated by the foil’s excellent thermal conductivity [62]. Moreover, explants are directly exposed to LN during cooling [16]. This method offers numerous advantages, including avoidance of explant manipulation during foil strip placement in the cryovial, which minimizes the risk of mechanical damage. By placing the explant directly into a drop of vitrification solution on the foil for dehydration, the exposure time to PVS2, which exhibits toxic properties, was significantly reduced. This method also ensures that the integrity of the explant is maintained and results in a high percentage of regeneration after thawing [62,63,64]. However, there are disadvantages, including the requirement for precise handling and extended time. Technical skills are required of the staff to ensure that the explants are not subjected to extensive exposure to PVS2 and at the same time, are not damaged or lost during handling or the application or removal of the vitrification solution [63,64]. 

### 2.6. Cryo-Plate Techniques

Recent advancements in cryopreservation have also centered around cryo-plates. The V cryo-plate method was developed by Yamamoto et al. in 2011 [65]. It combines encapsulation–vitrification and droplet vitrification methods using an aluminum cryo-plate with ten microwells. On the other hand, in 2013, Niino et al. introduced the D cryo-plate technique [66]. This method combines encapsulation and dehydration. There is a significant difference between the two protocols in regards to the time of treatment with the PVS solution. For the V cryo-plate method, the time is approximately four times shorter [66]. However, the D cryo-plate method, which uses physical dehydration, eliminates the risk of chemical stress and genetic alteration [38,67,68]. Depending on the species, its effectiveness is similar to or highly variable from that of other methods [69,70].

### 2.7. Cryo-Mesh Method

Funnekotter et al. developed the latest method, employing a stainless-steel cryo-mesh, in 2017 [71]. This method is analogous to the V-cryo plate method. The cryo-mesh facilitates rapid cooling and heating, provides uniform exposure to cryoprotectants, and minimizes the risk of mechanical damage to explants [71]. This approach offers a practical solution for the cryopreservation of fragile and tiny plant tissues.

Modern cryopreservation combines innovative and classical techniques to offer a wide range of solutions tailored to the specific needs of different plant species while minimizing the risk of damage and toxicity (Table 1). Despite the variety of available methods, the key challenges regarding controlling freezing conditions and optimizing protocols for individual species and tissues remain important for further research and technological improvement.

## 3. Cold Acclimatization 

As mentioned previously, the cryopreservation procedures indicate that plant explants are subjected to many stressors during their application, with temperature being a principal contributing factor, alongside dehydration and chemical toxicity. Furthermore, oxidative and osmotic stress, disturbed ionic homeostasis, and disorders associated with changes in the cells’ physical and metabolic properties must also be considered [72]. Consequently, damage to plant explants occurs at the cellular level, leading to vacuolization, cell membrane rupture (including the nuclear membrane), cell lysis, and autophagy [73]. Therefore, it can be inferred that tolerance to freezing is linked to tolerance to all of these factors. 

Plants native to temperate climates exhibit natural tolerance to freezing, as they undergo physiological and biochemical adaptation to cope with low temperatures and become dormant during winter [74]. This process, called cold acclimatization, is triggered after exposure to low but non-freezing temperatures [75]. Upon reception of a cold signal by plant cells, a plethora of physiological and metabolic alterations ensue, aiming to impede the formation of ice crystals within the cells. These alterations encompass modifications in the membrane lipid composition and the accumulation of cryoprotective molecules, such as soluble sugars, proteins, and other molecules that help stabilize cell structures and membranes. Substantial changes at the transcriptomic level are necessary for these alterations to manifest [76]. This adaptive capacity is not observed in plants from tropical and subtropical regions because they lack the genetic and physiological mechanisms to withstand freezing temperatures. Consequently, the cryopreservation of these plants is more challenging, yet not entirely impossible [64]. With the growing need for cryobank development, understanding the molecular basis associated with cryopreservation tolerance, demonstrated by the ability to survive and remain stable during storage, has become increasingly crucial.

In numerous cryopreservation procedures, plant material is subjected to cold pre-treatment, which enhances survival and regeneration following thawing. Cold acclimation pre-treatments stimulate a plant’s intrinsic defense mechanisms against the adverse effects of low temperatures [77]. This increases the content of starch grains, lipid bodies, sugars, dry matter, and phospholipids [78,79]. Changes in membrane lipid levels and composition may prevent freezing injury. After a relatively short acclimation period, improvements in cold tolerance have been observed in whole plants [78]. The beneficial impact of cold pre-treatment on donor plants has been documented in woody species, including *Malus x domestica*, *Pyrus communis*, and *Morus* [35,80,81,82]. Furthermore, the application of cold treatment has been proven to enhance the effectiveness of cryopreservation in plants derived from *in vitro* cultures, leading to improved survival rates in crops such as *Solanum tuberosa*, *Humulus lupulus*, and *Allium sativum* [83,84,85]. The benefits of cold pre-treatment have also been noted in isolated plant organs, such as apical meristems, in a range of species, including *Vaccinium*, *Rubus*, *Pyrus*, *Tanacetum cinerariifolium*, and *Phoenix dactylifera* [65,86,87,88,89]. Cold pre-treatment has also been beneficial for the cryopreservation of embryogenic calli [90,91]. Despite the documented benefits, the effectiveness of cold pre-treatment is not uniform across all plant species or even within different genotypes of the same species [21,92]. This variability presents a significant challenge in standardizing cryopreservation protocols, as the optimal conditions for cold pre-treatment, such as temperature, duration, and the specific developmental stage of the tissue, can vary widely between species. For example, while certain species may exhibit enhanced freezing tolerance following cold pre-treatment, others may not respond favorably or may even experience detrimental effects, emphasizing the need for species-specific optimization [93].

These findings highlight the significance of cold acclimatization as a critical factor contributing to freezing tolerance in numerous plant species. With the growing need for cryobank development, understanding the molecular basis associated with cryopreservation tolerance, demonstrated by the ability to survive and remain stable during storage, becomes increasingly crucial.

## 4. Molecular Basis of Freezing Tolerance

### 4.1. ICE–CBF–COR Cascade

Low temperatures induce both cold acclimation and dormancy. Thus, the two processes are interconnected, triggering the same cold-responsive (COR) pathway [94,95]. This pathway is part of the *inducer of CBF expression—C-repeat binding factors—cold-regulated* genes (*ICE–CBF–COR*) cascade [96,97]. The *ICE–CBF–COR* cascade represents a pivotal regulatory pathway in plant cold acclimation and freezing tolerance. This complex signaling cascade comprises three principal components that function synergistically to protect plants from cold stress [97]. Several physiological and biochemical changes are associated with cold acclimation, enhancing the freezing tolerance of plants following exposure to non-freezing temperatures.

#### 4.1.1. Cold-Responsive (COR) Genes 

Low temperatures induce both cold acclimation and dormancy. Thus, the two processes are interconnected, triggering the same cold responsive (COR) pathway [94,95]. This pathway is a part of the *inducer of CBF expression—C-repeat binding factors—cold-regulated* genes (*ICE–CBF–COR*) cascade [96,97]. Several physiological and biochemical changes are associated with cold acclimation, enhancing the freezing tolerance of plants following exposure to non-freezing temperatures. The COR pathway has been extensively studied in *Arabidopsis*, and over 200 *COR* genes have been identified [98]. However, this pathway is highly conserved among perennial plant species [95]. The *COR* pathway includes *low-temperature induced* (*LTI*), *late embryogenesis proteins* (*LEA*), *ABA-inducible protein-coding* (*KIN1* and *KIN2*), *responsive to desiccation* (*RD*), and *early dehydration-inducible* (*ERD*) genes [99,100,101,102,103]. These genes are responsible for accumulating cytoprotective proteins, including antifreeze proteins, chaperones, functional proteins, kinases, and osmoregulators, including soluble sugars [104,105,106,107]. This results in increased freezing tolerance, achieved by stabilizing the osmotic potential of cells and repairing cold-damaged membranes [108]. The promoters of *COR* genes contain conserved C-repeat (CRT/DRE) cis-elements to which C-repeat binding factors (CBFs) bind [109]. Most known *COR* genes exhibit a typical structural pattern, with two flanking exons in the 5′ untranslated region (UTR) and 3′ UTR, as well as a central intron [110]. Specific *COR* genes play an essential role in maintaining the stability of various biomolecules, such as membrane phospholipids, proteins, and cytoplasmic proteins. This is achieved by regulating hydrophobic interactions, maintaining ion homeostasis, and scavenging ROS, depending on the prevailing temperature range, i.e., pre-hardening, hardening, or plant recovery [111,112,113]. 

#### 4.1.2. CBF

The highly interconnected *COR* regulatory network is controlled by CBFs, which function as master transcription factors and are designated as *dehydration-responsive element-binding proteins* (*DREBs*) (Table 2) [114]. *CBFs* have been identified in many plant species and are members of the *APETALA2/ethylene response factor* (*AP2/ERF*) transcription factor family, one of the largest found in plants [115]. To ensure optimal plant growth, *CBF* expression must be maintained at a minimal level, without cold stress. However, following exposure to cold, *CBFs* are induced by transcription factors within 15 min, subsequently leading to the activation of downstream *COR* genes. CBFs regulate the majority of cold-responsive targets by binding to the DRE/CRT cis-element in the promoters of *COR* genes containing a conserved sequence (CCGAC) [116,117]. Prior research has demonstrated that individual proteins within the *CBF* family possess additional functions. For instance, in *Arabidopsis*, *CBF4* plays a role in drought tolerance, and *CBF2* functions as a negative regulator of *CBF1* and *CBF3* expression [118,119].

#### 4.1.3. ICE

Two motifs, i.e., ICEr1 and ICEr2, which are essential for cold induction, have been identified in the promoters of *CBFs* [120]. These are the binding sites for the *CBF expression 1–2* (*ICE1-2*) inducer, which encodes the MYC-type bHLH family of transcription factors. These proteins contain an essential DNA-binding domain and a helix–loop–helix motif that facilitates dimerization with other bHLH proteins. In addition to *CBFs*, ICE1 binds to the promoters of other *COR* genes, including *galactinol synthase 3* (GolS3), *KIN2*, *RCI2A*, *RCI2B*, and *COR413IM1*. This direct regulation ensures a robust cold response via the activation of multiple pathways [121].

*ICEs* MYC transcription factors are activated by cold-dependent post-translational modifications [122]. Previous studies show that *ICE1* is regulated by the high expression of *osmotically responsive gene 1* (*HOS1*), which has a ubiquitin E3 ligase function and is a cold-induced negative regulator of *CBFs* and thus, *COR* [123]. In response to low temperatures, HOS1 is transported to the nucleus, where it ubiquitinates ICE1, thereby directing this transcription factor to proteasomal degradation [124]. Cold-activated *open stomata kinase 1* (*OST1*) inhibits ICE1 degradation, which HOS1 mediates. It phosphorylates ICE1 and enhances its stability and transcriptional activity under cold stress conditions [125]. Moreover, *ICE1* interacts with the transcription factor MYB15, leading to a decrease in its expression. MYB15 has been demonstrated to bind to the promoter regions of *CBFs,* thereby suppressing their expression and consequently, negatively regulating freezing tolerance in plants. These observations suggest that HOS1, ICE1, and MYB15 operate in a cascade to modulate *CBFs* expression, thereby controlling cold acclimatization [126]. Furthermore, the stabilization of ICE1 by SIZ1 has been demonstrated to affect *CBF* expression. SIZ1 is a SUMO E3 ligase that facilitates the conjugation of small ubiquitin-related modifiers (SUMO) with protein substrates, thereby serving as a post-translational regulator that reduces freezing tolerance [127]. 

### 4.2. Other TFs Regulating CBFs

Additionally, other transcription factors that regulate the expression of CBFs have been identified, including calmodulin-binding transcription activator 3 (CAMTA3), CESTA (CES), brassinazole-resistant 1/brassinosteroid insensitive 1-EMS-suppressor 1 (BZR1/BES1), circadian clock-associated 1/late elongated hypocotyl (CCA1/LHY), phytochrome-interacting factors (PIFs), ethylene insensitive 3 (EIN3), and suppressor of constant overexpression 1 (SOC1) [107,128,129,130,131,132]. 

#### 4.2.1. CAMTA

*CAMTA* and Ca^2+^-regulated transcription factors have been shown to play a role in cold acclimation. *CAMTA3* and other *CAMTA* family members (*CAMTA1* and *CAMTA2*) undergo rapid induction in response to low temperatures. It has been demonstrated that CAMTA proteins can bind directly to the promoters of *CBF* genes (in particular, *CBF1* and *CBF2*), thereby facilitating their transcription. This binding is crucial for the cold-induced accumulation of *CBF* transcripts, leading to *COR* genes expression that enhances frost tolerance in plants [133,134]. Although the precise mechanisms underlying *CAMTA3*-induced *CBF* transcription remain partially elucidated, CAMTA proteins are recognized as critical players in calcium signaling pathways [135]. Calcium ions (Ca^2+^) are perceived in response to cold stress, which leads to the activation of *CAMTA3*. This activation is postulated to link calcium signaling to the transcriptional regulation of *CBF*, thereby promoting cold acclimation. In particular, CAMTA3 has been shown to interact with specific DNA motifs, such as the rapid stress response element (RSRE), in the promoters of *COR* genes, thereby increasing their expression under cold conditions [135,136]. *CAMTA* and Ca^2+^-regulated transcription factors have been shown to play a role in cold acclimation, whereby they induce the expression of *CBF*s. However, the precise mechanism of action remains unclear [135]. CAMTA proteins are highly conserved across species. They are present in various organisms, from mosses to flowering plants. They have been identified in more than 40 plant species [137].

#### 4.2.2. CCA1 and LHY

The transcription factors CCA1 and LHY are integral components of the circadian clock in plants and play an essential role in cold acclimation through the induction of *CBF* gene expression. CCA1 and LHY also induce the expression of *CBF* genes at low temperatures. Consequently, the expression of *COR* genes is enhanced, leading to increased cold tolerance [138]. The two transcription factors, CCA1 and LHY, possess the MYB motif and are involved in the circadian clock [139]. They positively regulate the expression of *CBF1*, *CBF2*, and *CBF3*. Genetic studies have revealed that plants with mutations in both genes (the *cca1-11/lhy-21* double mutant) display a markedly reduced capacity to induce *CBF* genes in response to cold temperatures. This leads to a notable decline in *COR* expression and a subsequent reduction in freezing tolerance [138]. CCA1 and LHY cooperate with other cold-responsive elements, including ICE1 and CAMTA3, to enhance *CBF* expression in response to cold stress. As the temperature decreases, CCA1 and LHY accumulate at the *CBF* loci, facilitating their transcription. This interaction is particularly effective in the morning, when CCA1 and LHY levels are elevated, facilitating robust CBF activation. In contrast, as the evening progresses, *CBF* levels decline, leading to reduced *CBF* expression and an attenuated response to cold stress [138]. Their participation in cold acclimation and freezing tolerance has been documented in several plant species, including *Arabidopsis, Oryza sativa*, and *Brassica oleracea* [138,140,141]. 

#### 4.2.3. SOC1

The *SOC1* gene, encoding a MADS-box transcription factor, is a negative regulator of the expression of *CBF* genes. SOC1 binds to various forms of the CArG box located in the distal and proximal regions of *CBFs*, thereby inhibiting the transcription of *CBF* genes and negatively regulating the cold response [142]. Evidence for the direct repression of *CBF* by *SOC1* is based on microarray analyses that have identified several *COR* genes (including *COR15a*, *COR15b*, *KIN1*, and *KIN2*) as targets of SOC1 regulation. In transgenic plants overexpressing *SOC1*, the transcription of these *COR* genes was significantly reduced. Conversely, loss-of-function mutations in *SOC1* increased the transcription of these genes when they were exposed to cold conditions [142,143]. Research findings have demonstrated that *SOC1* affects the kinetics of cold-inducible gene induction. To illustrate, experiments in which plants were exposed to cold temperatures revealed that *SOC1* mutants exhibited a more pronounced induction of *COR* genes than that observed in wild-type plants. This indicates that SOC1 plays a pivotal role in mitigating the cold-induced expression of these genes, thus preventing excessive activation of the cold stress response, which can be deleterious to plant growth and development [142]. Notably, the negative regulation of *CBF*s by *SOC1* is not significantly influenced by circadian rhythms, although the amplitude of its expression may exhibit variability. This suggests that *SOC1* consistently regulates the cold response, regardless of the time of day, which is crucial for plants that experience temperature fluctuations. The ability of *SOC1* to modulate *CBF*s expression in a circadian rhythm-independent manner highlights its significance in maintaining homeostasis during periods of low-temperature stress [142].

#### 4.2.4. PIFs

PIFs represent a subfamily of basic helix–loop–helix (bHLH) transcription factors that play significant and multifaceted roles in plant responses to environmental stress, particularly in regards to cold acclimation, and have been shown to play both beneficial and detrimental roles in this process [144,145]. They are primarily known for their involvement in light signaling pathways. However, their dual role in regulating the cold stress response, mainly through their interaction with CBFs, demonstrates the complexity of their involvement in plant adaptation mechanisms [146,147]. PIFs, particularly PIF3, PIF4, and PIF7, have been identified as negative regulators of *CBF* gene expression under cold stress conditions. They bind directly to the *CBF1*, *CBF2*, and *CBF3* promoter regions, thereby repressing transcription [144,145]. Therefore, it is evident that the repression of these pathways is crucial for preventing excessive activation of the cold acclimation pathway, which can lead to deleterious effects on growth and development. For example, under long-day conditions, PIF4 and PIF7 act as transcriptional repressors, thereby modulating the plant response to cold by controlling *CBF* expression. This mechanism allows plants to maintain growth while responding to cold stress, when necessary [147]. The regulatory function of PIFs in cold acclimation is also closely associated with their interaction with phytochrome B (phyB), a photoreceptor that mediates the response to light. Under unstressed conditions, *phyB* facilitates the degradation of PIFs, thereby relieving their inhibition of CBFs and enabling their acclimation to low temperatures. Conversely, in the presence of low temperatures, the stability of PIFs increases, resulting in their prolonged repression of *CBF*s expression. This dynamic balance between PIFs and phyB prevents plants from over-activating their responses to cold stress under non-stressful conditions, thereby optimizing their growth and development [145]. Notably, in response to cold stress, the interaction between CBF and PIF3 can stabilize phyB, a plant thermosensor. This stabilization enables phyB to regulate the expression of *COR* genes while concurrently suppressing the expression of *PIF1*, *PIF4*, and *PIF5*, which are associated with reduced cold tolerance. Consequently, the CBF-PIF3-phyB regulatory module is pivotal in mediating plant responses to cold stress, facilitating a balanced approach to stress adaptation and growth regulation [145].

#### 4.2.5. CESTA and BZR1/BES1

CESTA (CES) is a basic helix–loop–helix brassinosteroid (BR)-dependent transcription factor that facilitates the constitutive expression of *CBF*s. The presence of brassinosteroids has been demonstrated to enhance CES’s capacity to stimulate *CBF* transcription, which is vital for activating downstream *COR* genes that assist plants in tolerating low temperatures. CES directly interacts with the promoter regions of *CBF* genes, including *CBF1*, *CBF2*, and *CBF3*, thereby enhancing their transcription during periods of cold stress. This binding event is crucial for the rapid induction of *CBF*, which occurs in response to low temperatures and represents a pivotal aspect of cold acclimation [148,149]. CES operates with other BR signaling pathway components, including BZR1/BES1. These transcription factors have also been identified as critical regulators of *CBF*s expression via binding to *CBF* promoters [150]. This observation lends further credence to a coordinated BR signaling network hypothesized to enhance cold tolerance [149]. Moreover, during the cold acclimation process, BR- and CES-dependent *COR* genes are regulated independently of the *CBFs* [148]. Furthermore, evidence suggests that BZR1/BES1 plays a role in upregulating *CBFs* expression within the BR signaling pathway [150].

#### 4.2.6. EIN3

EIN3 is a critical transcription factor that mediates the ethylene signaling pathway in plants. It is pivotal in regulating *CBF* expression and modulating plant responses to cold stress [151]. *EIN3* functions as a negative regulator of *CBF* expression within the ethylene signaling pathway [131]. It directly binds to the promoters of CBF genes, thereby inhibiting their transcription [152,153]. The suppression of *CBF* by *EIN3* indicates that ethylene may play a role in modulating plants’ response to cold stress. Ethylene signaling, mediated by *EIN3,* could prioritize growth over stress resistance under certain conditions [154]. The ethylene pathway presents a contrasting scenario, wherein *EIN1* suppresses *CBFs* expression [131]. 

### 4.3. MAPK Cascades

The mitogen-activated protein kinase (MAPK) cascade also regulates cold stress responses through a series of phosphorylation events dependent on Ca^2+^ influx into the cytosol [155]. In response to exposure to low temperatures, specific MAPK kinases, including MPK3, MPK4, and MPK6, undergo rapid activation and play pivotal roles in mediating cold responses [156]. The activation of MPK3 and MPK6 results in the phosphorylation of ICE1 and its subsequent degradation, ultimately reducing *CBF* expression and hypersensitivity to freezing. Therefore, the MKK4/5–MPK3/6 cascade negatively regulates the cold response. *Mpk3* and *mpk6* mutants exhibit elevated *CBF* gene expression and enhanced freezing tolerance [156]. It suggests that the normal function of these MAPKs is to suppress *CBF* expression during cold stress. This negative regulation is vital to prevent the over-activation of the cold acclimation pathway, which can harm plant growth and development. In contrast, the MEKK1–MKK2–MPK4 pathway positively affects cold response [110]. This pathway suppresses MPK3 and MPK6 activities, allowing for controlled *CBF* expression [110]. The interactions between these diverse MAPK pathways underscore the complex nature of the signaling networks involved in cold acclimation. This results in enhanced expression of CBF2 and subsequently, *COR* genes [155]. One component of this cascade is the transcription factor CAMTA3 [157].

**Figure 1:**
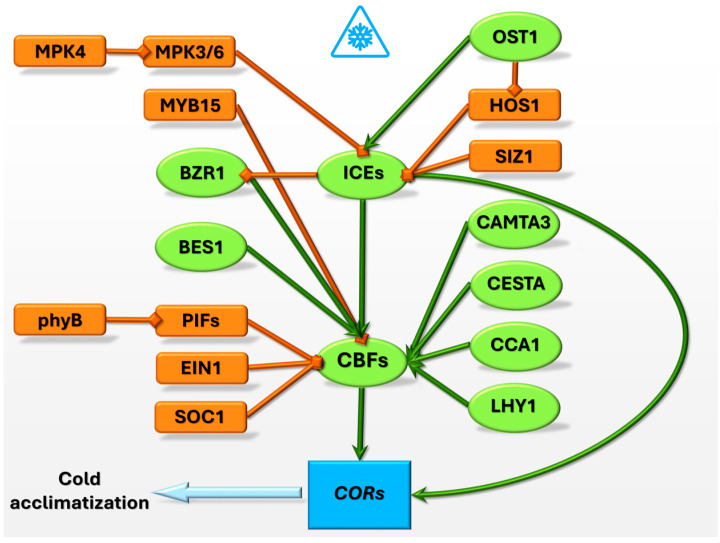
Cold response regulatory pathway. Green lines indicate positive regulation; orange lines indicate negative regulation.

The *ICE–CBF–COR* cascade is a critical regulatory mechanism that enables plants to adapt to cold stress and enhances freezing tolerance. Plants undergo physiological and biochemical changes through the coordinated action of ICE, CBF transcription factors, and *COR* genes (Figure 1). Protective proteins that stabilize cellular structures are produced. The involvement of other transcription factors, such as CAMTA3 and PIFs, highlights the complexity of this signaling pathway in the balance between cold tolerance and growth (Table 2). Overall, this signaling network is essential for cold acclimation. It ensures the survival and proper functioning of plants under low-temperature conditions.

**Table 2:** Transcription factors involved in cold acclimation.

Transcription Factor	Effect on Cold Acclimatization	Species	References
CBFs (DREBs)	positive regulator	*A. thaliana* *Z. mays* *O. sativa*	[114,115,116,117,118,119,158,159]
ICE1/ICE2	positive regulator	*A. thaliana*	[120,121,122]
HOS1	negative regulator	*A. thaliana*	[123,124]
OST1	positive regulator	*A. thaliana*	[125]
MYB15	negative regulator	*A. thaliana*	[126]
SIZ1	negative regulator	*A. thaliana*	[127]
CAMTA3	positive regulator	*A. thaliana*	[133,134,135,136,137]
CCA1/LHY	positive regulator	*A. thaliana* *O. sativa* *B. oleracea*	[138,139,140,141]
SOC1	negative regulator	*A. thaliana*	[142,143]
PIFs	positive/negative regulator	*A. thaliana*	[144,145,146,147]
CESTA	positive regulator	*A. thaliana*	[148,149]
BZR1/BES1	positive regulator	*A. thaliana*	[150]
EIN3	negative regulator	*A. thaliana*	[131,151,152,153,154]

### 4.4. Non-Coding RNAs

Non-coding RNAs (ncRNAs) constitute a family of RNAs that lack the protein-coding capacity. They generally assist in plant responses to environmental stressors and serve a pivotal function in regulating cold acclimatization and freezing tolerance [160]. They also play a crucial role in the pathways that regulate cold acclimation and freezing tolerance. The current classification of ncRNAs is based on transcript length and distinguishes between two broad categories: short non-coding RNAs (sRNAs) and long non-coding RNAs (lncRNAs).

#### 4.4.1. lncRNAs

Long non-coding RNAs constitute a highly diverse group of transcripts exceeding 200 nucleotides in length, with nuclear and cytoplasmic localization. They lack the protein-coding capacity but act as riboregulators, influencing gene expression through transcriptional and post-transcriptional regulation [161].

The involvement of lncRNAs in regulating cold adaptation has been demonstrated; however, their mechanisms of action remain unclear [162]. Many lncRNAs are expressed in response to cold treatment [163,164]. Targets for lncRNAs are the genes encoding *CBFs, LEAs*, and *WRKY* transcription factors, among others [165]. *SVALKA* is one of the most extensively studied lncRNAs and provides a mechanism for tightly regulating *CBF1.* The transcription of *SVALKA* by RNA polymerase II results in a cryptic lncRNA that overlaps with *CBF1* on the antisense strand called a*sCBF1*. The transcription of a*sCBF1* leads to a polymerases II collision, which immediately restricts the production of full-length transcripts of *CBF1*. The *SVALKA*-*asCBF1* cascade represents the mechanism that effectively regulates *CBF1* expression [166]. In contrast, *cold-induced lncRNA1* (*CIL*) has been identified as a positive regulator of cold stress in *Arabidopsis*. It regulates genes involved in reactive oxygen species (ROS) homeostasis, hormone signal transduction, and glucose metabolism, ultimately enhancing the tolerance of plants to cold [167]. Similarly, intergenic lncRNA *XH123*, described in cotton, has been demonstrated to function as a positive regulator of cold tolerance [168]. In the model plant *Medicago truncatula*, two lncRNAs, *MtCIR1* and *MtCIR2*, are associated with the regulatory network of cold tolerance by regulating *CBF* genes. The functional characterization of *MtCIR2*, located in the *CBF* gene, demonstrated that it induces the accumulation of sugars and reduces polysaccharide content within the hemicellulose of the cell wall [169]. Moreover, lncRNAs have been identified in cassava and have been demonstrated to function as positive regulators of cold tolerance. *Cold-responsive intergenic lncRNA 1* (*CRIR1*) regulates several cold-stress-related genes in a CBF-independent pathway, including *abscisic stress-ripening protein* (*ASR*) and *galactinol synthase* (*GOLS*), as well as the transcription factors *MeNAC* and *MeNFYA*. Additionally, *CRIR1* has been observed to interact with the cold shock protein 5 (MeCSP5), which has been demonstrated to function as an RNA chaperone [170]. CSP proteins have been shown to prevent the formation of mRNA secondary structures at low temperatures, thereby facilitating translation [171]. Consequently, *CRIR1* regulates the expression of *COR* genes and enhances their translational efficiency [170].

#### 4.4.2. sRNAs

Small regulatory RNA molecules (sRNAs) are pivotal in regulating gene expression, primarily at the post-transcriptional level. Among them, microRNAs (miRNAs) deserve particular attention. As expected, they regulate low-temperature response, cold acclimation, and freezing tolerance. miRNAs regulate gene expression at the post-transcriptional level through the post-transcriptional degradation or translational repression of target messenger RNAs (mRNAs) [172]. In addition, they can induce epigenetic modifications, including DNA and histone methylation [173]. A considerable number of miRNAs have been shown to alter their expression in response to cold temperatures [174,175,176]. In *Arabidopsis*, the CBF-dependent cold tolerance signaling pathway is positively regulated by *miR156*, *miR397*, and *miR394* [177,178,179]. However, in *O. sativa*, *OsmiR319* acts as a positive regulator of *CBFs*, whereas *OsmiR535* exerts a negative effect [180,181]. The upregulation of *miR408* has been demonstrated to enhance low-temperature tolerance in *Arabidopsis* [182]. In response to cold stress, the downregulation of *miR398* has been observed, leading to an increase in the expression of Cu/Zn superoxide dismutase (SOD), which detoxifies reactive oxygen species (ROS) in *Triticum aestivum*, *Solanum lycopersicum*, and *Vitis vinifera* [183,184,185]. Rice-specific *OsmiR1425* has been demonstrated to regulate cold tolerance by modulating the levels of pentatricopeptide repeat (PPR) protein [186,187]. Overexpression of miR402, which targets the *demeter-like protein 3*(*DML3*) gene (i.e., 5-methylcytosine DNA glycosidase), a key enzyme involved in the control of DNA methylation status, has been observed to positively influence the *Arabidopsis* response under low-temperature conditions [174]. Cold-induced *miR165/166* expression downregulation in *Arabidopsis* enhances cold and drought tolerance [188]. The upregulation of *miR528* has been observed to enhance chilling tolerance in several plant species, including *Arabidopsis*, *Pinus elliottii*, and *O. sativa*. This phenomenon is thought to result from the indirect inhibition of *MYB30* expression. MYB30, in turn, interacts with the β-amylase promoter, resulting in the repression of genes within the *BMY* family involved in the regulation of starch metabolism. This leads to the increased production of maltose, sucrose, and fructose [189]. It has been suggested that altering the expression levels of the microRNAs (miRNAs) associated with cold-induced genes is critical for successful cryopreservation [190].

### 4.5. Other Epigenetic Mechanisms

In addition to ncRNAs, the epigenetic regulation of cold stress is also influenced by mechanisms such as DNA methylation and histone modifications. In general, the term “DNA methylation” describes the transfer of a methyl group to the C5 position of a cytosine, which results in the formation of 5-methylcytosine. DNA methylation affects both the promoter regions of genes and their coding regions [191]. Modifying the DNA methylation pattern is a significant mechanism by which gene expression is regulated in response to cold treatment [192]. A reduction in total methylation levels was observed following cold treatment, indicating that cold-responsive genes in genotypes with increased tolerance to this abiotic factor may exhibit a higher potential for activation [193,194]. It has been demonstrated that both methylation and demethylation occur during cold adaptation. A total of 51 genes that exhibited both altered methylation levels and changes in expression following exposure to cold temperatures were identified in the rice genome. This group of genes includes those belonging to the *ICE–CBF–COR* pathway. One notable observation was the reduction in methylation at the promoter region of the *open stomatal 1* (*OST1*) gene homolog, which interacts with and phosphorylates ICE1 and upregulates its expression [195]. Additionally, cold treatment induces demethylation and the elevated transcriptional activity of the *ICE1* and *CBF2* genes. This occurs in conjunction with the demethylation of the promoters of genes linked to DNA methylation and the subsequent induction of their expression [196,197,198,199]. The chromatin remodeler *PICKLE* (*PKL*) has also been shown to play a pivotal role in cold stress responses in *Arabidopsis* [200]. Furthermore, *PKL* functions in collaboration with *photoperiod-independent early flowering 1* (*PIE1*), a *SWR1* chromatin remodeling complex member, to deposit H3K27me3 histones at gene loci [201]. The application of cold reduced the levels of H3K27me3 histones in *COR15* and *galactinol synthase 3* (*GOLS3*) genes. This suggests that *PKL* modulates the response to cold stress by modifying histones in *COR* genes [202]. In addition, the promoters of several *COR* genes, including *COR15* and *COR47*, are enriched for histone acetylation [203,204,205]. In *O. sativa* and *Z. mays*, histone acetylation is also induced in the *DREB1* promoter [206,207]. Moreover, the deacetylation of lysine residues on histone subunits H3 and H4 and an increase in the presence of the non-canonical H3 subunit acetylated at the ninth lysine residue (H3K9ac) were observed in *Z. mays*. A decrease in the DNA methylation and dimethylation of H3K9 accompanied this reaction [206,208].

### 4.6. Antifreeze Proteins (AFPs)

Antifreeze proteins (AFPs) are specialized proteins, glycopeptides, and peptides that play a crucial role in the cryopreservation of plant tissues by binding to ice and inhibiting its formation and recrystallization, thereby protecting cells from freezing damage. In plants, AFPs have been identified in species that experience freezing conditions, in which they bind to the surface of ice crystals, thereby inhibiting their growth and preventing the formation of large, potentially damaging ice crystals [209]. As uncontrolled ice formation can lead to mechanical damage to cell structures, dehydration, and cell death, this ice-binding activity is essential during the cryopreservation process.

The principal mechanism by which AFPs protect plant cells consists of their interaction with the ice nuclei. AFPs adsorb on the surface of tiny ice crystals, thereby reducing the freezing point of water, a phenomenon known as thermal hysteresis. This binding effectively prevents the growth of ice crystals by creating a barrier that inhibits the addition of water molecules to the ice network [210]. Moreover, AFPs can modify the ice crystal morphology, facilitating the formation of smoother, less damaging shapes. This reduces the risk of physical damage to cell membranes and organelles [211]. It is especially beneficial during cryopreservation because it preserves the integrity of the cellular structure, which is critical for the viability of the tissues at the time of thawing.

In plants, the most common types of AFP proteins are pathogenesis-related (PR) proteins [212]. Chitinase-like proteins have been identified in several species, including *Secale cereale*, *Solanum dulcamara*, *Chimonanthus praecox*, *Bromus inermis*, *Hevea brasiliensis*, and *Picea abies* [213,214,215,216,217,218]. While thaumatin-like IBPs have been identified in *T. aestivum*, β-1,3 glucanase, and chitinase-like IBPs have been observed in *Raphanus sativus* [219,220,221]. Furthermore, IBPs displaying structural homology with polygalacturonase inhibitor proteins (PGIPs) were identified in *Daucus carota* and *Hippophaes rhamnoideum* [222,223,224]. In addition to identifying PR proteins in plants, other types of AFPs have been documented. Among these, STHP-64 has been identified in *Solanum dulcamara*, revealing sequence homology with the transcription factor WRKY [225]. Some dehydrins also exhibit AFP functions. For example, PCA60 has been identified in *Prunus persica* [226]. AFPs are efficiently secreted into the apoplastic space. Therefore, they are detected in the growth medium rather than in cells cultured *in vitro* [215,227].

### 4.7. Late Embryogenesis Abundant (LEA) Proteins

Late embryogenesis abundant (LEA) proteins are a group of proteins that play critical roles in plant tissue survival during cold and freezing exposure. They were first identified during the embryo maturation and desiccation processes [228]. LEAs are crucial stress-responsive proteins. They accumulate in response to desiccation, cold, and osmotic stress commonly encountered during cryopreservation [229]. Dehydrins (DHNs) belonging to the LEA family play a role in the anti-aggregation of enzymes and in protecting cell structures during cold stress and dehydration [230]. They are highly hydrophilic and intrinsically disordered, which allows them to remain soluble in a dehydrated state and to interact with various cellular components to protect them from damage [231,232]. Critical to their protective function during the freezing and thawing processes of cryopreservation is their ability to stabilize the cellular matrix in a glassy state, as well as proteins, membranes, and other structures under stress [232,233]. DHNs belong to the LEA II group and protect plant cells from dehydration damage caused by drought, cold, salinity, and osmotic stress [234,235]. During freezing, ice formation in the extracellular spaces leads to a decrease in the water potential. It causes water to move out of the cell, resulting in cellular dehydration. Dehydration can lead to membrane phase transitions, causing membranes to leak or fuse improperly, which can be fatal to cells. Dehydrins help prevent these deleterious effects by binding to membrane phospholipids, stabilizing the membrane structure, and preventing phase transitions that lead to leakage [236,237,238]. In addition, dehydrins can interact with lipid bilayers to form a protective layer around the membrane. This mitigates the effects of cold-induced dehydration [239]. Numerous DHNs associated with the cold response have been identified, including DHN24, P-80, LTI30, WCS120, and WCOR410 [236,240,241,242].

### 4.8. Chaperones

Molecular chaperones are essential proteins that assist in the folding, assembling, and stabilizing of other proteins, particularly under stress conditions. They are, therefore, critical for plant cryopreservation. Chaperones are a family of unrelated proteins that bind specifically to the surface of other proteins. This prevents the formation of non-functional structures resulting from abnormal interactions [243]. Chaperones include heat shock proteins (HSPs), which stabilize proteins and membranes. In addition, HSPs can participate in protein refolding [244]. Based on molecular weight, amino acid sequence homology, and function, five major classes of HSPs have been identified in plants. These classes include HSP100, HSP90, HSP70, HSP60, and the small heat shock proteins (sHSPs) [245,246]. Levels of HSP70, HSP90, chaperonin 20 (HSP60), and HSP17.4C1 (sHSP) are increased in *Arabidopsis* upon exposure to low temperatures. The overexpression of specific chaperones in genetically modified plants has increased their tolerance to cryogenic stress and survival after cryopreservation [247,248,249,250]. This group also includes RNA chaperones ubiquitous in all living organisms. They facilitate the proper folding of RNA molecules during RNA metabolism. In *Arabidopsis*, the RNA chaperone AtCSP2 plays a role in adapting to cold and future development [251]. In addition, the glycine-rich RNA-binding proteins AtGRP2 and AtGRP7 have been observed to provide cold and freezing tolerance to plants. They also exhibit RNA chaperone activity during cold adaptation [252,253].

### 4.9. Osmoprotectants

In response to low temperatures, plants accumulate a variety of soluble compounds. These include free sterols, sterol glucosides, acylated sterols, glucosides, arabinoxylans, cerebrosides, raffinose and other soluble sugars, amino acids (alanine, glycine, proline, and serine), polyamines, and betaines [254]. Raffinose family oligosaccharides (RFOs), including raffinose and stachyose, accumulate in dormant tissues. They provide tolerance to cold and drought stress [255]. In *Castanea sativa*, it has been shown that low temperature induces the upregulation of dual-specificity protein phosphatase 4 (DSP4). DSP4 most likely increases oligosaccharide synthesis during winter dormancy through starch dephosphorylation and degradation [256]. Genes encoding galactinol synthase (GolS), which catalyzes the first step in synthesizing RFOs, are upregulated in the dormant buds of woody perennials [257,258]. T. aestivum membrane sterols are essential in mitigating plant responses to low temperatures [259]. In response to abiotic stress, proline plays a role in osmotic regulation, as well as in the stabilization of membrane proteins, the induction of stress gene expression, and the scavenging of ROS. It also regulates cytosolic acidity, maintains the NAD/NADH ratio, increases photosystem II photochemical efficiency, and reduces lipid peroxidation [260]. Increased endogenous proline levels in response to cold stress have been observed in species that exhibit natural cold tolerance. The stabilization of transcriptional and translational mechanisms is likely facilitated by glycine betaine. There is also a correlation between its levels and cold tolerance [261].

## 5. Challenges and Future Directions

Cryopreservation offers significant potential for the long-term conservation of plant genetic resources. However, the natural diversity of species, with unique physiological, biochemical, and genetic characteristics, represents a significant challenge in standardizing cryopreservation protocols. It has been observed that some species, and even genotypes within a species, exhibit greater resilience to cryopreservation procedures, suggesting a reduced probability of survival following freezing and thawing. Notably, a lack of tolerance to cryopreservation is widespread among tropical and subtropical species with no natural tolerance to cold and desiccation. Moreover, the tissue’s developmental stage and physiological state at the time of cryopreservation significantly impact viability following thawing. Immature or unacclimated tissues are typically more vulnerable to damage. This highlights the necessity for precise timing and pre-treatment strategies, such as cold acclimation or the application of osmotic stress prior to cryopreservation. In contrast, the effective vitrification of tissues without producing the side effects associated with cytotoxicity represents a significant challenge, particularly in the case of large and complex explants such as buds, meristems, or embryos. Achieving consistent and reproducible results is also a significant challenge, particularly when scaling up protocols for implementation in gene banks or conservation programs. Variable and unpredictable cryopreservation efficiencies are influenced by several factors, including the genetic makeup of the material, handling of explants, expertise of the personnel involved, and equipment available in the cryopreservation laboratory.

In the future, the further development and integration of omics technologies with cryopreservation research can revolutionize the field by providing a holistic view of the biological processes involved. By combining data from genomics, transcriptomics, proteomics, and metabolomics, scientists can construct comprehensive plant cryotolerance models, leading to the identification of critical regulatory networks and molecular pathways. This system’s biological approach can uncover new targets for genetic engineering or chemical intervention, paving the way for developing next-generation cryopreservation techniques. Furthermore, the prediction of cryopreservation outcomes based on specific genetic, proteomic, or metabolic profiles could be facilitated by applying bioinformatics, machine learning, and deep neural networks to omics data. Predictive models can be developed to assess the cryotolerance of plant tissues before cryopreservation, allowing for the selection of the most appropriate methods and treatments for each species or genotype. Implementing such a customized approach to cryopreservation could significantly improve its efficiency.

## 6. Conclusions

Cryopreservation is an essential technique for the long-term conservation of plant genetic resources, and it provides a viable solution for preserving plant diversity in the face of global environmental challenges. This process depends on understanding and optimizing the molecular mechanisms underlying plant freezing tolerance. Key factors contributing to successful cryopreservation include cold acclimation, which enhances the survival of plant tissues during freezing, and the regulation of cold-responsive genes via pathways such as the *ICE–CBF–COR* signaling cascade. Transcription factors, non-coding RNAs, and epigenetic modifications refine the ability of plants to withstand cryogenic conditions.

However, the effectiveness of cryopreservation varies across species and genotypes, particularly in tropical and subtropical plants that lack natural cold tolerance. This variability highlights the need for species-specific protocols and pre-treatment strategies. The integration of advanced omics technologies promises to address these challenges by providing a comprehensive understanding of the biological processes involved, potentially leading to more effective and standardized cryopreservation methods.

In summary, cryopreservation is a powerful tool for plant conservation. However, its success depends on continued research and technological advances to overcome limitations and ensure its broad application to diverse plant species.

## Figures and Tables

**Table 1:** Summary of cryopreservation techniques for the conservation of plant genetic resources.

Protocol	Description	Advantages	Disadvantages	Applications	References
Slow Freezing	Employs a gradual cooling rate (up to 2 °C per minute) followed by immersion in LN. Prevents intracellular ice formation.	Effective for winter dormant buds, shoot tips, and cell suspensions; low concentration of cryoprotectants; standardized procedures.	Requires costly programmable freezers and precise cryoprotectant regulation. Toxicity issues with some plant tissues.	Conservation of plant genetic resources, particularly for temperate and subtropical plants.	[18,24,25,26,28,31]
Cryopreservation of Dormant Buds	A protocol based on slow freezing, applicable to trees and shrubs in temperate climates that undergo natural dormancy.	No *in vitro* stage, reducing process length, infection risk, and costs. No toxic cryoprotectants.	Requires a programmable freezer. Different success rates across genotypes within a species due to variations in freeze tolerance.	Preservation of trees and shrubs from temperate climates.	[16,27,33,34,36,37]
Vitrification	Employs rapid cooling of biological material treated with cryoprotectants to prevent ice crystal formation by transforming liquid into a glassy state.	Extensive applicability to a diverse range of species and tissues. Cost-effective, simple, and minimal equipment requirements.	High toxicity of cryoprotectants.	Cryopreservation of shoot tips, somatic and zygotic embryos, and other plant tissues.	[28,40,42,43,44,45]
Encapsulation Dehydration	The explants are encapsulated in calcium alginate gel beads, dehydrated, and then cryopreserved in LN.	Low toxicity compared to that of other methods; protects explants from mechanical damage; adaptable to different species.	Labor- and time-consuming process. Requires precise control of dehydration.	Widely applicable across species; used for the cryopreservation of various explants.	[49,50,51,52,53]
Encapsulation–vitrification	Combines encapsulation dehydration with vitrification, providing protection during cryopreservation and reducing osmotic stress and cryoprotectant toxicity.	Allows cryopreservation without the need for programmable freezers; applicable to tropical and subtropical species.	Cytotoxicity; technical complexity.	Cryopreservation of explants, particularly from tropical or subtropical species.	[54,55,56,57,58]
Droplet Vitrification	Small explants are placed in a droplet of vitrification solution on aluminum foil and rapidly cooled by LN.	Reduces exposure to toxic cryoprotectants; minimizes mechanical damage; maintains explant integrity.	Requires precise handling and technical skill to avoid overexposure to cryoprotectants and mechanical damage.	Cryopreservation of apical meristems, shoot tips, and other small explants.	[59,60,61,63,64]
Cryo-plate Techniques	Uses aluminum cryo-plates with microwells for encapsulation–vitrification (V cryo-plate) or encapsulation dehydration (D cryo-plate).	Rapid cooling and reduced chemical stress in D cryo-plate method.	Varying effectiveness, depending on species.	Applicable to a wide range of plant tissues, depending on the species.	[65,66,67,68,69,70]
Cryo-mesh Method	Uses stainless-steel cryo-mesh for rapid cooling and heating, providing uniform exposure to cryoprotectants and minimizing mechanical damage.	Practical for fragile and tiny plant tissues.	The precision of this method requires considerable attention to detail in the manipulation of the sample.	Cryopreservation of fragile and tiny plant tissues.	[71]

## References

[1] Ceballos G., Ehrlich P.R., Barnosky A.D., García A., Pringle R.M., Palmer T.M. (2015). Accelerated modern human–induced species losses: Entering the sixth mass extinction. Sci. Adv..

[2] McCauley D.J., Pinsky M.L., Palumbi S.R., Estes J.A., Joyce F.H., Warner R.R. (2015). Marine defaunation: Animal loss in the global ocean. Science.

[3] Barnosky A.D., Matzke N., Tomiya S., Wogan G.O., Swartz B., Quental T.B., Marshall C., McGuire J.L., Lindsey E.L., Maguire K.C. (2011). Has the Earth’s sixth mass extinction already arrived?. Nature.

[4] Cowie R.H., Bouchet P., Fontaine B. (2022). The Sixth Mass Extinction: Fact, fiction or speculation?. Biol. Rev..

[5] Lu Y., Yang Y., Sun B., Yuan J., Yu M., Stenseth N.C., Bullock J.M., Obersteiner M. (2020). Spatial variation in biodiversity loss across China under multiple environmental stressors. Sci. Adv..

[6] Singh V., Shukla S., Singh A. (2021). The principal factors responsible for biodiversity loss. Open J. Plant Sci..

[7] Corlett R.T. (2016). Plant diversity in a changing world: Status, trends, and conservation needs. Plant Divers..

[8] Mahanayak B. (2024). Ex-situ and in-situ conservation of wild life. World J. Biol. Pharm. Health Sci..

[9] Hawkes J.G., Maxted N., Ford-Lloyd B.V. (2012). The Ex Situ Conservation of Plant Genetic Resources.

[10] Mounce R., Smith P., Brockington S. (2017). Ex situ conservation of plant diversity in the world’s botanic gardens. Nat. Plants.

[11] Pence V.C., Ballesteros D., Walters C., Reed B.M., Philpott M., Dixon K.W., Pritchard H.W., Culley T.M., Vanhove A.-C. (2020). Cryobiotechnologies: Tools for expanding long-term ex situ conservation to all plant species. Biol. Conserv..

[12] Benelli C. (2021). Plant Cryopreservation: A Look at the Present and the Future. Plants.

[13] Nagel M., Pence V., Ballesteros D., Lambardi M., Popova E., Panis B. (2024). Plant cryopreservation: Principles, applications, and challenges of banking plant diversity at ultralow temperatures. Annu. Rev. Plant Biol..

[14] Bartels-Rausch T., Bergeron V., Cartwright J.H., Escribano R., Finney J.L., Grothe H., Gutiérrez P.J., Haapala J., Kuhs W.F., Pettersson J.B. (2012). Ice structures, patterns, and processes: A view across the icefields. Rev. Mod. Phys..

[15] Kartha K., Engelmann F. (1994). Cryopreservation and germplasm storage. Plant Cell and Tissue Culture.

[16] Benelli C., De Carlo A., Engelmann F. (2013). Recent advances in the cryopreservation of shoot-derived germplasm of economically important fruit trees of Actinidia, Diospyros, Malus, Olea, Prunus, Pyrus and Vitis. Biotechnol. Adv..

[17] Benito M.G., Clavero-Ramírez I., López-Aranda J. (2004). The use of cryopreservation for germplasm conservation of vegetatively propagated crops. Span. J. Agric. Res..

[18] Ruta C., Lambardi M., Ozudogru E.A. (2020). Biobanking of vegetable genetic resources by in vitro conservation and cryopreservation. Biodivers. Conserv..

[19] Normah M., Sulong N., Reed B.M. (2019). Cryopreservation of shoot tips of recalcitrant and tropical species: Advances and strategies. Cryobiology.

[20] Höfer M., Hanke M.-V. (2017). Cryopreservation of fruit germplasm. Vitr. Cell. Dev. Plant.

[21] Panta A., Panis B., Ynouye C., Swennen R., Roca W., Tay D., Ellis D. (2015). Improved cryopreservation method for the long-term conservation of the world potato germplasm collection. Plant Cell Tissue Organ Cult. (PCTOC).

[22] Harding K., Johnston J.W., Benson E.E. (2009). Exploring the physiological basis of cryopreservation success and failure in clonally propagated in vitro crop plant germplasm. Agric. Food Sci..

[23] Sakai A. (1956). Survival of plant tissue of super-low temperatures. Low Temp. Sci. B.

[24] Mazur P. (1984). Freezing of living cells: Mechanisms and implications. Am. J. Physiol. Cell Physiol..

[25] Engelmann F. (2004). Plant cryopreservation: Progress and prospects. Vitr. Cell. Dev. Plant.

[26] Reed B.M., Dumet D., Denoma J.M., Benson E.E. (2001). Validation of cryopreservation protocols for plant germplasm conservation: A pilot study using *Ribes* L.. Biodivers. Conserv..

[27] Towill L., Ellis D., Reed B.M. (2008). Cryopreservation of dormant buds. Plant Cryopreservation: A Practical Guide.

[28] Reed B.M., Uchendu E. (2008). Plant Cryopreservation: A Practical Guide.

[29] Reed B., Chang Y. (1997). Medium and long-term storage of in vitro cultures of temperate fruit and nut crops. Conservation of Plant Genetic Resources In Vitro.

[30] Ozudogru E.A., Lambardi M. (2016). Cryotechniques for the long-term conservation of embryogenic cultures from woody plants. In Vitro Embryogenesis in Higher Plants.

[31] Engelmann F., Takagi H. (2000). Cryopreservation of Tropical Plant Germplasm: Current Research Progress and Applications.

[32] Best B.P. (2015). Cryoprotectant toxicity: Facts, issues, and questions. Rejuvenation Res..

[33] Sakai A. (1960). Survival of the Twig of Woody Plants at −196 °C. Nature.

[34] Forsline P.L., Towill L.E., Waddell J.W., Stushnoff C., Lamboy W.F., McFerson J.R. (1998). Recovery and longevity of cryopreserved dormant apple buds. J. Am. Soc. Hortic. Sci..

[35] Höfer M. (2015). Cryopreservation of winter-dormant apple buds: Establishment of a duplicate collection of Malus germplasm. Plant Cell Tissue Organ Cult. (PCTOC).

[36] Vogiatzi C., Grout B., Wetten A., Toldam-Andersen T. (2011). Cryopreservation of winter-dormant apple buds: I-variation in recovery with cultivar and winter conditions. CryoLetters.

[37] Panis B., Nagel M., van Den Houwe I. (2020). Challenges and prospects for the conservation of crop genetic resources in field genebanks, in in vitro collections and/or in liquid nitrogen. Plants.

[38] Wang M.-R., Chen L., Teixeira da Silva J.A., Volk G.M., Wang Q.-C. (2018). Cryobiotechnology of apple (Malus spp.): Development, progress and future prospects. Plant Cell Rep..

[39] Volk G., Jenderek M., Chao C. Prioritization of Malus accessions for collection cryopreservation at the USDA-ARS National Center for Genetic Resources Preservation. Proceedings of the XIV EUCARPIA Symposium on Fruit Breeding and Genetics 1172.

[40] Langis R., Steponkus P. (1991). Vitrification of isolated rye protoplasts: Protection against dehydration injury by ethylene glycol. Cryo Lett..

[41] Sakai A., Kobayashi S., Oiyama I. (1990). Cryopreservation of nucellar cells of navel orange (*Citrus sinensis* Osb. var. *Brasiliensis tanaka*) by vitrification. Plant Cell Rep..

[42] Möller J., Gutzow I., Schmelzer J.W. (2006). Freezing-in and production of entropy in vitrification. J. Chem. Phys..

[43] Grout B. (1995). Introduction to the in vitro preservation of plant cells, tissues and organs. Genetic Preservation of Plant Cells In Vitro.

[44] Benson E.E. (1999). Cryopreservation. Plant Conservation Biotechnology.

[45] Leopold A.C. (1986). Membranes, Metabolism, and Dry Organisms.

[46] Senula A., Keller E., Sanduijav T., Yohannes T. (2007). Cryopreservation of cold-acclimated mint (*Mentha* spp.) shoot tips using a simple vitrification protocol. Cryo Lett..

[47] Yamada T., Sakai A., Matsumura T., Higuchi S. (1991). Cryopreservation of apical meristems of white clover (*Trifolium repens* L.) by vitrification. Plant Sci..

[48] Mikuła A., Tomiczak K., Domżalska L., Rybczyński J.J. (2015). Cryopreservation of Gentianaceae: Trends and applications. The Gentianaceae-Volume 2: Biotechnology and Applications.

[49] Fabre J. (1990). Encapsulation Dehydration—A new approach to cryopreservation of Solanum shoot-tips. Cryo Lett..

[50] Redenbaugh K., Paasch B.D., Nichol J.W., Kossler M.E., Viss P.R., Walker K.A. (1986). Somatic seeds: Encapsulation of asexual plant embryos. Bio/technology.

[51] Engelmann F., Arnao M.-T.G., Wu Y., Escobar R. (2008). Development of encapsulation dehydration. Plant Cryopreservation: A Practical Guide.

[52] Gonzalez-Arnao M., Engelmann F., Urra C., Morenza M., Rios A. (1998). Cryopreservation of citrus apices using the encapsulation-dehydration technique. Cryo Lett..

[53] Gonzalez-Arnao M.T., Engelmann F. (2006). Cryopreservation of plant germplasm using the encapsulation-dehydration technique: Review and case study on sugarcane. CryoLetters.

[54] Tannoury M., Ralambosoa J., Kaminski M., Dereuddre J. (1991). Cryopreservation by vitrification of coated shoot-tips of carnation (*Dianthus caryophyllus* L.) cultured in vitro. Comptes Rendus L’académie Sci..

[55] Wang Q., Perl A. (2006). Cryopreservation of embryogenic cell suspensions by encapsulation-vitrification. Plant Cell Culture Protocols.

[56] Kaviani B., Dehkaei M.P., Hashemabadi D., Darabi A. (2010). Cryopreservation of Lilium ledebourii (Baker) Bioss by encapsulation-vitrification and in vivo media for planting of germplasms. Am.-Eur. J. Agric. Environ. Sci..

[57] Moges A.D., Shibli R.A., Karam N.S. (2004). Cryopreservation of African violet (Saintpaulia ionantha Wendl.) shoot tips. Vitr. Cell. Dev. Plant.

[58] Yin M., Hong S. (2009). Cryopreservation of Dendrobium candidum Wall. ex Lindl. protocorm-like bodies by encapsulation-vitrification. Plant Cell Tissue Organ Cult. (PCTOC).

[59] Pennycooke J., Towill L. (2000). Cryopreservation of shoot tips from in vitro plants of sweet potato [*Ipomoea batatas* (L.) Lam.] by vitrification. Plant Cell Rep..

[60] Kartha K., Leung N., Mroginski L. (1982). In vitro growth responses and plant regeneration from cryopreserved meristems of cassava (Manihot esculenta Crantz). Z. Pflanzenphysiol..

[61] Panis B., Piette B., Swennen R. (2005). Droplet vitrification of apical meristems: A cryopreservation protocol applicable to all Musaceae. Plant Sci..

[62] Kulus D., Zalewska M. (2014). Cryopreservation as a tool used in long-term storage of ornamental species—A review. Sci. Hortic..

[63] Wang M.-R., Lambardi M., Engelmann F., Pathirana R., Panis B., Volk G.M., Wang Q.-C. (2021). Advances in cryopreservation of in vitro-derived propagules: Technologies and explant sources. Plant Cell Tissue Organ Cult. (PCTOC).

[64] Kaviani B., Kulus D. (2022). Cryopreservation of endangered ornamental plants and fruit crops from tropical and subtropical regions. Biology.

[65] Yamamoto S.-I., Rafique T., Priyantha W.S., Fukui K., Matsumoto T., Niino T. (2011). Development of a cryopreservation procedure using aluminium cryo-plates. CryoLetters.

[66] Niino T., Yamamoto S.-I., Fukui K., Martínez C.R.C., Arizaga M.V., Matsumoto T., Engelmann F. (2013). Dehydration improves cryopreservation of mat rush (Juncus decipiens Nakai) basal stem buds on cryo-plates. CryoLetters.

[67] Harding K. (2004). Genetic integrity of cryopreserved plant cells: A review. CryoLetters.

[68] Wang B., Wang R.-R., Cui Z.-H., Bi W.-L., Li J.-W., Li B.-Q., Ozudogru E.A., Volk G.M., Wang Q.-C. (2014). Potential applications of cryogenic technologies to plant genetic improvement and pathogen eradication. Biotechnol. Adv..

[69] Vujović T., Chatelet P., Ružić Đ., Engelmann F. (2015). Cryopreservation of *Prunus* spp. using aluminium cryo-plates. Sci. Hortic..

[70] Engelmann-Sylvestre I., Engelmann F. (2015). Cryopreservation of in vitro-grown shoot tips of Clinopodium odorum using aluminium cryo-plates. Vitr. Cell. Dev. Plant.

[71] Funnekotter B., Bunn E., Mancera R.L. (2017). Cryo-mesh: A simple alternative cryopreservation protocol. CryoLetters.

[72] Ren L., Zhang D., Chen G.-Q., Reed B.M., Shen X.-H., Chen H.-Y. (2015). Transcriptomic profiling revealed the regulatory mechanism of Arabidopsis seedlings response to oxidative stress from cryopreservation. Plant Cell Rep..

[73] Whelehan L.M., Funnekotter B., Bunn E., Mancera R.L. (2022). The case for studying mitochondrial function during plant cryopreservation. Plant Sci..

[74] Juurakko C.L., Walker V.K. (2021). Cold acclimation and prospects for cold-resilient crops. Plant Stress.

[75] Téoulé E., Géry C. (2020). Mapping of Quantitative Trait Loci (QTL) Associated with Plant Freezing Tolerance and Cold Acclimation. Plant Cold Acclimation: Methods and Protocols.

[76] Liu Y., Cai Y., Li Y., Zhang X., Shi N., Zhao J., Yang H. (2022). Dynamic changes in the transcriptome landscape of Arabidopsis thaliana in response to cold stress. Front. Plant Sci..

[77] Reed B. (1996). Pretreatment strategies for cryopreservation of plant tissues. In Vitro Conservation of Plant Genetic Resources.

[78] Itzhaki H., Pauls K., Borochov A. (1991). Effects of cold hardening on microsomal membrane properties and phosphatidylcholine biosynthesis in canola (*Brassica napus*) leaves. J. Plant Physiol..

[79] Tanino K., Weiser C.J., Fuchigami L.H., Chen T.H. (1990). Water content during abscisic acid induced freezing tolerance in bromegrass cells. Plant Physiol..

[80] Chang Y., Reed B.M. (2000). Extended alternating-temperature cold acclimation and culture duration improve pear shoot cryopreservation. Cryobiology.

[81] Niino T., Sakai A. (1992). Cryopreservation of alginate-coated in vitro-grown shoot tips of apple, pear and mulberry. Plant Sci..

[82] Seibert M., Wetherbee P.J. (1977). Increased survival and differentiation of frozen herbaceous plant organ cultures through cold treatment. Plant Physiol..

[83] Reed B.M., Okut N., D’Achino J., Narver L., DeNoma J. (2003). Cold storage and cryopreservation of hops (*Humulus* L.) shoot cultures through application of standard protocols. CryoLetters.

[84] Keller E. (2005). Improvement of cryopreservation results in garlic using low temperature preculture and high-quality in vitro plantlets. CryoLetters.

[85] Kaczmarczyk A., Rokka V.-M., Keller E.J. (2011). Potato shoot tip cryopreservation. A review. Potato Res..

[86] Fki L., Bouaziz N., Chkir O., Benjemaa-Masmoudi R., Rival A., Swennen R., Drira N., Panis B. (2013). Cold hardening and sucrose treatment improve cryopreservation of date palm meristems. Biol. Plant..

[87] Reed B. (1989). Effect of cold hardening and cooling rate on the survival of apical meristems of vaccinium species frozen in liquid nitrogen. Cryo Lett..

[88] Reed B. (1988). Cold acclimation as a method to improve survival of cryopreserved Rubus meristems. Cryo Lett..

[89] Reed B.M. (1993). Responses to ABA and cold acclimation are genotype dependent for cryopreserved blackberry and raspberry meristems. Cryobiology.

[90] Lambardi M., Ozudogru E.A., Benelli C. (2008). Cryopreservation of embryogenic cultures. Plant Cryopreservation: A Practical Guide.

[91] Häggman H.M., Ryynänen L.A., Aronen T.S., Krajnakova J. (1998). Cryopreservation of embryogenic cultures of Scots pine. Plant Cell Tissue Organ Cult..

[92] Sales E., Nebauer S.G., Arrillaga I., Segura J. (2001). Cryopreservation of Digitalis obscura selected genotypes by encapsulation-dehydration. Planta Medica.

[93] Ballesteros D., Martínez M.T., Sanchez-Romero C., Montalban I.A., Sales E., Moncalean P., Arrillaga I., Corredoira E. (2024). Current status of the cryopreservation of embryogenic material of woody species. Front. Plant Sci..

[94] Welling A., Moritz T., Palva E.T., Junttila O. (2002). Independent activation of cold acclimation by low temperature and short photoperiod in hybrid aspen. Plant Physiol..

[95] Wingler A. (2015). Comparison of signaling interactions determining annual and perennial plant growth in response to low temperature. Front. Plant Sci..

[96] Bremer A., Kent B., Hauß T., Thalhammer A., Yepuri N.R., Darwish T.A., Garvey C.J., Bryant G., Hincha D.K. (2017). Intrinsically disordered stress protein COR15A resides at the membrane surface during dehydration. Biophys. J..

[97] Guo J., Ren Y., Tang Z., Shi W., Zhou M. (2019). Characterization and expression profiling of the ICE-CBF-COR genes in wheat. PeerJ.

[98] Li X., Liu C., Zhao Z., Ma D., Zhang J., Yang Y., Liu Y., Liu H. (2020). COR27 and COR28 are novel regulators of the COP1–HY5 regulatory hub and photomorphogenesis in Arabidopsis. Plant Cell.

[99] Aslam M., Fakher B., Ashraf M.A., Cheng Y., Wang B., Qin Y. (2022). Plant low-temperature stress: Signaling and response. Agronomy.

[100] Ding Y., Shi Y., Yang S. (2019). Advances and challenges in uncovering cold tolerance regulatory mechanisms in plants. New Phytol..

[101] Yu Z., Wang X., Zhang L. (2018). Structural and functional dynamics of dehydrins: A plant protector protein under abiotic stress. Int. J. Mol. Sci..

[102] Kosová K., Klíma M., Prášil I.T., Vítámvás P. (2021). COR/LEA proteins as indicators of frost tolerance in Triticeae: A comparison of controlled versus field conditions. Plants.

[103] Li J., Liu Z., Hao X., Chang X., Zhao Z., Chen G., Hu W., Gao S., Huang Q. (2023). Low-Temperature Signaling Pathways and Their Signaling Factors in Plant. Agric. Sci..

[104] Liou Y.-C., Daley M.E., Graham L.A., Kay C.M., Walker V.K., Sykes B.D., Davies P.L. (2000). Folding and structural characterization of highly disulfide-bonded beetle antifreeze protein produced in bacteria. Protein Expr. Purif..

[105] Reddy A.R., Chaitanya K.V., Vivekanandan M. (2004). Drought-induced responses of photosynthesis and antioxidant metabolism in higher plants. J. Plant Physiol..

[106] Chandler J.W. (2018). Class VIIIb APETALA2 ethylene response factors in plant development. Trends Plant Sci..

[107] Shi Y., Ding Y., Yang S. (2018). Molecular regulation of CBF signaling in cold acclimation. Trends Plant Sci..

[108] Chinnusamy V., Zhu J., Zhu J.-K. (2007). Cold stress regulation of gene expression in plants. Trends Plant Sci..

[109] Leuendorf J.E., Frank M., Schmülling T. (2020). Acclimation, priming and memory in the response of Arabidopsis thaliana seedlings to cold stress. Sci. Rep..

[110] Hwarari D., Guan Y., Ahmad B., Movahedi A., Min T., Hao Z., Lu Y., Chen J., Yang L. (2022). ICE-CBF-COR signaling cascade and its regulation in plants responding to cold stress. Int. J. Mol. Sci..

[111] Welti R., Li W., Li M., Sang Y., Biesiada H., Zhou H.-E., Rajashekar C., Williams T.D., Wang X. (2002). Profiling membrane lipids in plant stress responses: Role of phospholipase Dα in freezing-induced lipid changes in Arabidopsis. J. Biol. Chem..

[112] Wang W., Vinocur B., Altman A. (2003). Plant responses to drought, salinity and extreme temperatures: Towards genetic engineering for stress tolerance. Planta.

[113] Okawa K., Nakayama K., Kakizaki T., Yamashita T., Inaba T. (2008). Identification and characterization of Cor413im proteins as novel components of the chloroplast inner envelope. Plant Cell Environ..

[114] Zhen Y., Ungerer M.C. (2008). Relaxed selection on the CBF/DREB1 regulatory genes and reduced freezing tolerance in the southern range of Arabidopsis thaliana. Mol. Biol. Evol..

[115] Agarwal P.K., Agarwal P., Reddy M., Sopory S.K. (2006). Role of DREB transcription factors in abiotic and biotic stress tolerance in plants. Plant Cell Rep..

[116] Liu Q., Kasuga M., Sakuma Y., Abe H., Miura S., Yamaguchi-Shinozaki K., Shinozaki K. (1998). Two transcription factors, DREB1 and DREB2, with an EREBP/AP2 DNA binding domain separate two cellular signal transduction pathways in drought-and low-temperature-responsive gene expression, respectively, in Arabidopsis. Plant Cell.

[117] Gilmour S.J., Zarka D.G., Stockinger E.J., Salazar M.P., Houghton J.M., Thomashow M.F. (1998). Low temperature regulation of the Arabidopsis CBF family of AP2 transcriptional activators as an early step in cold-induced COR gene expression. Plant J..

[118] Haake V., Cook D., Riechmann J., Pineda O., Thomashow M.F., Zhang J.Z. (2002). Transcription factor CBF4 is a regulator of drought adaptation in Arabidopsis. Plant Physiol..

[119] Novillo F., Alonso J.M., Ecker J.R., Salinas J. (2004). CBF2/DREB1C is a negative regulator of CBF1/DREB1B and CBF3/DREB1A expression and plays a central role in stress tolerance in Arabidopsis. Proc. Natl. Acad. Sci. USA.

[120] Zarka D.G., Vogel J.T., Cook D., Thomashow M.F. (2003). Cold induction of Arabidopsis CBF genes involves multiple ICE (inducer of CBF expression) promoter elements and a cold-regulatory circuit that is desensitized by low temperature. Plant Physiol..

[121] Tang K., Zhao L., Ren Y., Yang S., Zhu J.K., Zhao C. (2020). The transcription factor ICE1 functions in cold stress response by binding to the promoters of CBF and COR genes. J. Integr. Plant Biol..

[122] Knight M.R., Knight H. (2012). Low-temperature perception leading to gene expression and cold tolerance in higher plants. New Phytol..

[123] Wang B., Duan C.G., Wang X., Hou Y.J., Yan J., Gao C., Kim J.H., Zhang H., Zhu J.K. (2015). HOS 1 regulates Argonaute1 by promoting transcription of the micro RNA gene MIR 168b in Arabidopsis. Plant J..

[124] Dong C.-H., Agarwal M., Zhang Y., Xie Q., Zhu J.-K. (2006). The negative regulator of plant cold responses, HOS1, is a RING E3 ligase that mediates the ubiquitination and degradation of ICE1. Proc. Natl. Acad. Sci. USA.

[125] Ding Y., Li H., Zhang X., Xie Q., Gong Z., Yang S. (2015). OST1 kinase modulates freezing tolerance by enhancing ICE1 stability in Arabidopsis. Dev. Cell.

[126] Agarwal M., Hao Y., Kapoor A., Dong C.-H., Fujii H., Zheng X., Zhu J.-K. (2006). A R2R3 type MYB transcription factor is involved in the cold regulation of CBF genes and in acquired freezing tolerance. J. Biol. Chem..

[127] Miura K., Jin J.B., Lee J., Yoo C.Y., Stirm V., Miura T., Ashworth E.N., Bressan R.A., Yun D.-J., Hasegawa P.M. (2007). SIZ1-mediated sumoylation of ICE1 controls CBF3/DREB1A expression and freezing tolerance in Arabidopsis. Plant Cell.

[128] Kidokoro S., Yoneda K., Takasaki H., Takahashi F., Shinozaki K., Yamaguchi-Shinozaki K. (2017). Different cold-signaling pathways function in the responses to rapid and gradual decreases in temperature. Plant Cell.

[129] Barrero-Gil J., Salinas J. (2017). CBFs at the crossroads of plant hormone signaling in cold stress response. Mol. Plant.

[130] Wang D.-Z., Jin Y.-N., Ding X.-H., Wang W.-J., Zhai S.-S., Bai L.-P., Guo Z.-F. (2017). Gene regulation and signal transduction in the *ICE–CBF–COR* signaling pathway during cold stress in plants. Biochemistry.

[131] Shi Y., Tian S., Hou L., Huang X., Zhang X., Guo H., Yang S. (2012). Ethylene signaling negatively regulates freezing tolerance by repressing expression of CBF and type-A ARR genes in Arabidopsis. Plant Cell.

[132] Al-Beyroutiová M., Sabo M., Sleziak P., Dušinský R., Birčák E., Hauptvogel P., Kilian A., Švec M. (2016). Evolutionary relationships in the genus *Secale* revealed by DArTseq DNA polymorphism. Plant Syst. Evol..

[133] Doherty C.J., Van Buskirk H.A., Myers S.J., Thomashow M.F. (2009). Roles for Arabidopsis CAMTA transcription factors in cold-regulated gene expression and freezing tolerance. Plant Cell.

[134] Abdullah S.N.A., Azzeme A.M., Yousefi K. (2022). Fine-tuning cold stress response through regulated cellular abundance and mechanistic actions of transcription factors. Front. Plant Sci..

[135] Chao L., Kim Y., Gilmour S.J., Thomashow M.F. (2022). Temperature modulation of CAMTA3 gene induction activity is mediated through the DNA binding domain. Plant J..

[136] Iqbal Z., Memon A.G., Ahmad A., Iqbal M.S. (2022). Calcium mediated cold acclimation in plants: Underlying signaling and molecular mechanisms. Front. Plant Sci..

[137] Rahman H., Yang J., Xu Y.-P., Munyampundu J.-P., Cai X.-Z. (2016). Phylogeny of plant CAMTAs and role of AtCAMTAs in nonhost resistance to Xanthomonas oryzae pv. oryzae. Front. Plant Sci..

[138] Dong M.A., Farré E.M., Thomashow M.F. (2011). Circadian clock-associated 1 and late elongated hypocotyl regulate expression of the C-repeat binding factor (CBF) pathway in Arabidopsis. Proc. Natl. Acad. Sci. USA.

[139] Park M.-J., Kwon Y.-J., Gil K.-E., Park C.-M. (2016). LATE ELONGATED HYPOCOTYL regulates photoperiodic flowering via the circadian clock in Arabidopsis. BMC Plant Biol..

[140] Lu X., Zhou Y., Fan F., Peng J., Zhang J. (2020). Coordination of light, circadian clock with temperature: The potential mechanisms regulating chilling tolerance in rice. J. Integr. Plant Biol..

[141] Song H., Yi H., Han C.-T., Park J.-I., Hur Y. (2018). Allelic variation in Brassica oleracea CIRCADIAN CLOCK ASSOCIATED 1 (BoCCA1) is associated with freezing tolerance. Hortic. Environ. Biotechnol..

[142] Seo E., Lee H., Jeon J., Park H., Kim J., Noh Y.-S., Lee I. (2009). Crosstalk between cold response and flowering in Arabidopsis is mediated through the flowering-time gene SOC1 and its upstream negative regulator FLC. Plant Cell.

[143] Lee J., Lee I. (2010). Regulation and function of SOC1, a flowering pathway integrator. J. Exp. Bot..

[144] Jiang B., Shi Y., Zhang X., Xin X., Qi L., Guo H., Li J., Yang S. (2017). PIF3 is a negative regulator of the CBF pathway and freezing tolerance in Arabidopsis. Proc. Natl. Acad. Sci. USA.

[145] Jiang B., Shi Y., Peng Y., Jia Y., Yan Y., Dong X., Li H., Dong J., Li J., Gong Z. (2020). Cold-induced CBF–PIF3 interaction enhances freezing tolerance by stabilizing the phyB thermosensor in Arabidopsis. Mol. Plant.

[146] Bian Y., Chu L., Lin H., Qi Y., Fang Z., Xu D. (2022). PIFs-and COP1-HY5-mediated temperature signaling in higher plants. Stress Biol..

[147] Kidokoro S., Maruyama K., Nakashima K., Imura Y., Narusaka Y., Shinwari Z.K., Osakabe Y., Fujita Y., Mizoi J., Shinozaki K. (2009). The phytochrome-interacting factor PIF7 negatively regulates DREB1 expression under circadian control in Arabidopsis. Plant Physiol..

[148] Eremina M., Unterholzner S.J., Rathnayake A.I., Castellanos M., Khan M., Kugler K.G., May S.T., Mayer K.F., Rozhon W., Poppenberger B. (2016). Brassinosteroids participate in the control of basal and acquired freezing tolerance of plants. Proc. Natl. Acad. Sci. USA.

[149] Planas-Riverola A., Gupta A., Betegón-Putze I., Bosch N., Ibañes M., Caño-Delgado A.I. (2019). Brassinosteroid signaling in plant development and adaptation to stress. Development.

[150] Li H., Ye K., Shi Y., Cheng J., Zhang X., Yang S. (2017). BZR1 positively regulates freezing tolerance via CBF-dependent and CBF-independent pathways in Arabidopsis. Mol. Plant.

[151] Dolgikh V.A., Pukhovaya E.M., Zemlyanskaya E.V. (2019). Shaping ethylene response: The role of EIN3/EIL1 transcription factors. Front. Plant Sci..

[152] Lado J., Rey F., Manzi M. (2023). Phytohormones and Cold Stress Tolerance. Plant Hormones and Climate Change.

[153] Robison J.D., Yamasaki Y., Randall S.K. (2019). The ethylene signaling pathway negatively impacts CBF/DREB-regulated cold response in soybean (Glycine max). Front. Plant Sci..

[154] Shi Y., Ding Y., Yang S. (2015). Cold signal transduction and its interplay with phytohormones during cold acclimation. Plant Cell Physiol..

[155] Teige M., Scheikl E., Eulgem T., Doczi R., Ichimura K., Shinozaki K., Dangl J.L., Hirt H. (2004). The MKK2 pathway mediates cold and salt stress signaling in Arabidopsis. Mol. Cell.

[156] Zhao F., Zheng Y.-F., Zeng T., Sun R., Yang J.-Y., Li Y., Ren D.-T., Ma H., Xu Z.-H., Bai S.-N. (2017). Phosphorylation of SPOROCYTELESS/NOZZLE by the MPK3/6 kinase is required for anther development. Plant Physiol..

[157] Jiang X., Hoehenwarter W., Scheel D., Lee J. (2020). Phosphorylation of the CAMTA3 transcription factor triggers its destabilization and nuclear export. Plant Physiol..

[158] Jaglo-Ottosen K.R., Gilmour S.J., Zarka D.G., Schabenberger O., Thomashow M.F. (1998). Arabidopsis CBF1 overexpression induces COR genes and enhances freezing tolerance. Science.

[159] Borba A.R., Serra T.S., Górska A., Gouveia P., Cordeiro A.M., Reyna-Llorens I., Kneřová J., Barros P.M., Abreu I.A., Oliveira M.M. (2018). Synergistic binding of bHLH transcription factors to the promoter of the maize NADP-ME gene used in C4 photosynthesis is based on an ancient code found in the ancestral C3 state. Mol. Biol. Evol..

[160] Jiang N., Cui J. (2023). Non-coding RNA assisted plant response to stresses. Front. Plant Sci..

[161] Bhogireddy S., Mangrauthia S.K., Kumar R., Pandey A.K., Singh S., Jain A., Budak H., Varshney R.K., Kudapa H. (2021). Regulatory non-coding RNAs: A new frontier in regulation of plant biology. Funct. Integr. Genom..

[162] Jha U.C., Nayyar H., Roychowdhury R., Prasad P.V., Parida S.K., Siddique K.H. (2023). Non-coding RNAs (ncRNAs) in plants: Master regulators for adapting to extreme temperature conditions. Plant Physiol. Biochem..

[163] Calixto C.P., Tzioutziou N.A., James A.B., Hornyik C., Guo W., Zhang R., Nimmo H.G., Brown J.W. (2019). Cold-dependent expression and alternative splicing of Arabidopsis long non-coding RNAs. Front. Plant Sci..

[164] Leng Y., Sun J., Wang J., Liu H., Zheng H., Zhang M., Zhao H., Zou D. (2020). Genome-wide lncRNAs identification and association analysis for cold-responsive genes at the booting stage in rice (*Oryza sativa* L.). Plant Genome.

[165] Wang P., Dai L., Ai J., Wang Y., Ren F. (2019). Identification and functional prediction of cold-related long non-coding RNA (lncRNA) in grapevine. Sci. Rep..

[166] Kindgren P., Ard R., Ivanov M., Marquardt S. (2018). Transcriptional read-through of the long non-coding RNA SVALKA governs plant cold acclimation. Nat. Commun..

[167] Liu G., Liu F., Wang Y., Liu X. (2022). A novel long noncoding RNA CIL1 enhances cold stress tolerance in Arabidopsis. Plant Sci..

[168] Cao Z., Zhao T., Wang L., Han J., Chen J., Hao Y., Guan X. (2021). The lincRNA XH123 is involved in cotton cold-stress regulation. Plant Mol. Biol..

[169] Zhao M., Tian R., Sun X., Zhang W.H. (2023). lncRNA MtCIR2 positively regulates plant-freezing tolerance by modulating CBF/DREB1 gene clusters. Plant Cell Environ..

[170] Li S., Cheng Z., Dong S., Li Z., Zou L., Zhao P., Guo X., Bao Y., Wang W., Peng M. (2022). Global identification of full-length cassava lncRNAs unveils the role of cold-responsive intergenic lncRNA 1 in cold stress response. Plant Cell Environ..

[171] Keto-Timonen R., Hietala N., Palonen E., Hakakorpi A., Lindström M., Korkeala H. (2016). Cold shock proteins: A minireview with special emphasis on Csp-family of enteropathogenic Yersinia. Front. Microbiol..

[172] Bartel D.P. (2004). MicroRNAs: Genomics, biogenesis, mechanism, and function. Cell.

[173] Liu Q., Chen Y.-Q. (2010). A new mechanism in plant engineering: The potential roles of microRNAs in molecular breeding for crop improvement. Biotechnol. Adv..

[174] Tiwari B., Habermann K., Arif M.A., Weil H.L., Garcia-Molina A., Kleine T., Mühlhaus T., Frank W. (2020). Identification of small RNAs during cold acclimation in Arabidopsis thaliana. BMC Plant Biol..

[175] Ouellet F., Charron J.-B. (2013). Cold acclimation and freezing tolerance in plants. Encycl. Life Sci..

[176] Chen H., Chen X., Chen D., Li J., Zhang Y., Wang A. (2015). A comparison of the low temperature transcriptomes of two tomato genotypes that differ in freezing tolerance: Solanum lycopersicum and Solanum habrochaites. BMC Plant Biol..

[177] Dong C.-H., Pei H. (2014). Over-expression of miR397 improves plant tolerance to cold stress in Arabidopsis thaliana. J. Plant Biol..

[178] Song J.B., Gao S., Wang Y., Li B.W., Zhang Y.L., Yang Z.M. (2016). miR394 and its target gene LCR are involved in cold stress response in Arabidopsis. Plant Gene.

[179] Zhao J., Shi M., Yu J., Guo C. (2022). SPL9 mediates freezing tolerance by directly regulating the expression of CBF2 in Arabidopsis thaliana. BMC Plant Biol..

[180] Sun M., Shen Y., Yang J., Cai X., Li H., Zhu Y., Jia B., Sun X. (2020). miR535 negatively regulates cold tolerance in rice. Mol. Breed..

[181] Wang S.-T., Sun X.-L., Hoshino Y., Yu Y., Jia B., Sun Z.-W., Sun M.-Z., Duan X.-B., Zhu Y.-M. (2014). MicroRNA319 positively regulates cold tolerance by targeting OsPCF6 and OsTCP21 in rice (*Oryza sativa* L.). PLoS ONE.

[182] Ma C., Burd S., Lers A. (2015). mi R 408 is involved in abiotic stress responses in A rabidopsis. Plant J..

[183] Cao X., Wu Z., Jiang F., Zhou R., Yang Z. (2014). Identification of chilling stress-responsive tomato microRNAs and their target genes by high-throughput sequencing and degradome analysis. BMC Genom..

[184] Sun X., Fan G., Su L., Wang W., Liang Z., Li S., Xin H. (2015). Identification of cold-inducible microRNAs in grapevine. Front. Plant Sci..

[185] Wang B., Sun Y.-F., Song N., Wei J.-P., Wang X.-J., Feng H., Yin Z.-Y., Kang Z.-S. (2014). MicroRNAs involving in cold, wounding and salt stresses in *Triticum aestivum* L.. Plant Physiol. Bioch..

[186] Jeong D.-H., Park S., Zhai J., Gurazada S.G.R., De Paoli E., Meyers B.C., Green P.J. (2011). Massive analysis of rice small RNAs: Mechanistic implications of regulated microRNAs and variants for differential target RNA cleavage. Plant Cell.

[187] Komori T., Imaseki H. (2005). Transgenic rice hybrids that carry the Rf-1 gene at multiple loci show improved fertility at low temperature. Plant Cell Environ..

[188] Yan J., Zhao C., Zhou J., Yang Y., Wang P., Zhu X., Tang G., Bressan R.A., Zhu J.-K. (2016). The miR165/166 mediated regulatory module plays critical roles in ABA homeostasis and response in Arabidopsis thaliana. PLoS Genet..

[189] Tang W., Thompson W.A. (2019). OsmiR528 enhances cold stress tolerance by repressing expression of stress response-related transcription factor genes in plant cells. Curr. Genom..

[190] Ekinci M.H., Kayıhan D.S., Kayıhan C., Özden Çiftçi Y. (2021). The role of microRNAs in recovery rates of Arabidopsis thaliana after short term cryo-storage. Plant Cell Tissue Organ Cult. (PCTOC).

[191] Lister R., O’Malley R.C., Tonti-Filippini J., Gregory B.D., Berry C.C., Millar A.H., Ecker J.R. (2008). Highly integrated single-base resolution maps of the epigenome in Arabidopsis. Cell.

[192] Thiebaut F., Hemerly A.S., Ferreira P.C.G. (2019). A role for epigenetic regulation in the adaptation and stress responses of non-model plants. Front. Plant Sci..

[193] Kumar G., Rattan U.K., Singh A.K. (2016). Chilling-mediated DNA methylation changes during dormancy and its release reveal the importance of epigenetic regulation during winter dormancy in apple (*Malus x domestica Borkh*.). PLoS ONE.

[194] Rakei A., Maali-Amiri R., Zeinali H., Ranjbar M. (2016). DNA methylation and physio-biochemical analysis of chickpea in response to cold stress. Protoplasma.

[195] Guo H., Wu T., Li S., He Q., Yang Z., Zhang W., Gan Y., Sun P., Xiang G., Zhang H. (2019). The Methylation Patterns and Transcriptional Responses to Chilling Stress at the Seedling Stage in Rice. Int. J. Mol. Sci..

[196] Tang X., Wang Q., Yuan H., Huang X. (2018). Chilling-induced DNA Demethylation is associated with the cold tolerance of Hevea brasiliensis. BMC Plant Biol..

[197] Liu T., Li Y., Duan W., Huang F., Hou X. (2017). Cold acclimation alters DNA methylation patterns and confers tolerance to heat and increases growth rate in Brassica rapa. J. Exp. Bot..

[198] Zhang B., Tieman D.M., Jiao C., Xu Y., Chen K., Fei Z., Giovannoni J.J., Klee H.J. (2016). Chilling-induced tomato flavor loss is associated with altered volatile synthesis and transient changes in DNA methylation. Proc. Natl. Acad. Sci. USA.

[199] Sicilia A., Scialò E., Puglisi I., Lo Piero A.R. (2020). Anthocyanin Biosynthesis and DNA Methylation Dynamics in Sweet Orange Fruit [*Citrus sinensis* L. (Osbeck)] under Cold Stress. J. Agric. Food Chem..

[200] Yang R., Hong Y., Ren Z., Tang K., Zhang H., Zhu J.-K., Zhao C. (2019). A role for PICKLE in the regulation of cold and salt stress tolerance in Arabidopsis. Front. Plant Sci..

[201] Carter B., Bishop B., Ho K.K., Huang R., Jia W., Zhang H., Pascuzzi P.E., Deal R.B., Ogas J. (2018). The Chromatin Remodelers PKL and PIE1 Act in an Epigenetic Pathway That Determines H3K27me3 Homeostasis in Arabidopsis. Plant Cell.

[202] Kwon C.S., Lee D., Choi G., Chung W.-I. (2009). Histone occupancy-dependent and -independent removal of H3K27 trimethylation at cold-responsive genes in Arabidopsis. Plant J..

[203] Zhu J., Jeong J.C., Zhu Y., Sokolchik I., Miyazaki S., Zhu J.-K., Hasegawa P.M., Bohnert H.J., Shi H., Yun D.-J. (2008). Involvement of *Arabidopsis* HOS15 in histone deacetylation and cold tolerance. Proc. Natl. Acad. Sci. USA.

[204] Pavangadkar K., Thomashow M.F., Triezenberg S.J. (2010). Histone dynamics and roles of histone acetyltransferases during cold-induced gene regulation in Arabidopsis. Plant Mol. Biol..

[205] Park J., Lim C.J., Shen M., Park H.J., Cha J.-Y., Iniesto E., Rubio V., Mengiste T., Zhu J.-K., Bressan R.A. (2018). Epigenetic switch from repressive to permissive chromatin in response to cold stress. Proc. Natl. Acad. Sci. USA.

[206] Hu Y., Zhang L., Zhao L., Li J., He S., Zhou K., Yang F., Huang M., Jiang L., Li L. (2011). Trichostatin A selectively suppresses the cold-induced transcription of the ZmDREB1 gene in maize. PLoS ONE.

[207] Roy D., Paul A., Roy A., Ghosh R., Ganguly P., Chaudhuri S. (2014). Differential acetylation of histone H3 at the regulatory region of OsDREB1b promoter facilitates chromatin remodelling and transcription activation during cold stress. PLoS ONE.

[208] Hu Y., Zhang L., He S., Huang M., Tan J., Zhao L., Yan S., Li H., Zhou K., Liang Y. (2012). Cold stress selectively unsilences tandem repeats in heterochromatin associated with accumulation of H3K9ac. Plant Cell Environ..

[209] Griffith M., Yaish M.W. (2004). Antifreeze proteins in overwintering plants: A tale of two activities. Trends Plant Sci..

[210] Knight C., Cheng C., DeVries A. (1991). Adsorption of alpha-helical antifreeze peptides on specific ice crystal surface planes. Biophys. J..

[211] Sirotinskaya V., Bar Dolev M., Yashunsky V., Bahari L., Braslavsky I. (2024). Extended Temperature Range of the Ice-Binding Protein Activity. Langmuir.

[212] Wisniewski M., Willick I.R., Duman J.G., Livingston D., Newton S.S., Ramløv H., Friis D.S. (2020). Plant Antifreeze Proteins. Antifreeze Proteins Volume 1: Environment, Systematics and Evolution.

[213] Hon W.-C., Griffith M., Mlynarz A., Kwok Y.C., Yang D.S. (1995). Antifreeze proteins in winter rye are similar to pathogenesis-related proteins. Plant Physiol..

[214] Duman J.G. (1994). Purification and characterization of a thermal hysteresis protein from a plant, the bittersweet nightshade Solanum dulcamara. Biochim. Biophys. Acta (BBA) Protein Struct. Mol. Enzymol..

[215] Nakamura T., Ishikawa M., Nakatani H., Oda A. (2008). Characterization of cold-responsive extracellular chitinase in bromegrass cell cultures and its relationship to antifreeze activity. Plant Physiol..

[216] Zhang S.-H., Wei Y., Liu J.-L., Yu H.-M., Yin J.-H., Pan H.-Y., Baldwin T. (2011). An apoplastic chitinase CpCHT1 isolated from the corolla of wintersweet exhibits both antifreeze and antifungal activities. Biol. Plant..

[217] Jarząbek M., Pukacki P., Nuc K. (2009). Cold-regulated proteins with potent antifreeze and cryoprotective activities in spruces (*Picea* spp.). Cryobiology.

[218] Martínez-Caballero S., Cano-Sánchez P., Mares-Mejía I., Díaz-Sánchez A.G., Macías-Rubalcava M.L., Hermoso J.A., Rodríguez-Romero A. (2014). Comparative study of two GH 19 chitinase-like proteins from Hevea brasiliensis, one exhibiting a novel carbohydrate-binding domain. FEBS J..

[219] Chun J., Yu X., Griffith M. (1998). Genetic studies of antifreeze proteins and their correlation with winter survival in wheat. Euphytica.

[220] Kontogiorgos V., Regand A., Yada R., Goff H.D. (2007). Isolation and characterization of ice structuring proteins from cold-acclimated winter wheat grass extract for recrystallization inhibition in frozen foods. J. Food Biochem..

[221] Kawahara H., Fujii A., Inoue M., Kitao S., Fukuoka J., Obata H. (2009). Antifreeze activity of cold acclimated Japanese radish and purification of antifreeze peptide. CryoLetters.

[222] Gupta R., Deswal R. (2014). Refolding of β-stranded class I chitinases of Hippophae rhamnoides enhances the antifreeze activity during cold acclimation. PLoS ONE.

[223] Meyer K., Keil M., Naldrett M.J. (1999). A leucine-rich repeat protein of carrot that exhibits antifreeze activity. FEBS Lett..

[224] Worrall D., Elias L., Ashford D., Smallwood M., Sidebottom C., Lillford P., Telford J., Holt C., Bowles D. (1998). A carrot leucine-rich-repeat protein that inhibits ice recrystallization. Science.

[225] Huang T., Duman J.G. (2002). Cloning and characterization of a thermal hysteresis (*antifreeze*) protein with DNA-binding activity from winter bittersweet nightshade, Solanum dulcamara. Plant Mol. Biol..

[226] Wisniewski M., Webb R., Balsamo R., Close T.J., Yu X.M., Griffith M. (1999). Purification, immunolocalization, cryoprotective, and antifreeze activity of PCA60: A dehydrin from peach (*Prunus persica*). Physiol. Plant..

[227] Takahashi D., Kawamura Y., Uemura M. (2013). Changes of detergent-resistant plasma membrane proteins in oat and rye during cold acclimation: Association with differential freezing tolerance. J. Proteome Res..

[228] Dure L., Greenway S.C., Galau G.A. (1981). Developmental biochemistry of cottonseed embryogenesis and germination: Changing messenger ribonucleic acid populations as shown by in vitro and in vivo protein synthesis. Biochemistry.

[229] Tunnacliffe A., Wise M.J. (2007). The continuing conundrum of the LEA proteins. Naturwissenschaften.

[230] Graether S.P., Boddington K.F. (2014). Disorder and function: A review of the dehydrin protein family. Front. Plant Sci..

[231] Shao H.-B., Liang Z.-S., Shao M.-A. (2005). LEA proteins in higher plants: Structure, function, gene expression and regulation. Colloids Surf. B Biointerfaces.

[232] Koubaa S., Bremer A., Hincha D.K., Brini F. (2019). Structural properties and enzyme stabilization function of the intrinsically disordered LEA_4 protein TdLEA3 from wheat. Sci. Rep..

[233] Hincha D.K., Zuther E., Popova A.V. (2021). Stabilization of Dry Sucrose Glasses by Four LEA_4 Proteins from Arabidopsis thaliana. Biomolecules.

[234] Abdul Aziz M., Sabeem M., Mullath S.K., Brini F., Masmoudi K. (2021). Plant Group II LEA Proteins: Intrinsically Disordered Structure for Multiple Functions in Response to Environmental Stresses. Biomolecules.

[235] Riyazuddin R., Nisha N., Singh K., Verma R., Gupta R. (2022). Involvement of dehydrin proteins in mitigating the negative effects of drought stress in plants. Plant Cell Rep..

[236] Danyluk J., Perron A., Houde M., Limin A., Fowler B., Benhamou N., Sarhan F. (1998). Accumulation of an Acidic Dehydrin in the Vicinity of the Plasma Membrane during Cold Acclimation of Wheat. Plant Cell.

[237] Szabala B.M., Fudali S., Rorat T. (2014). Accumulation of acidic SK3 dehydrins in phloem cells of cold- and drought-stressed plants of the Solanaceae. Planta.

[238] Heyen B.J., Alsheikh M.K., Smith E.A., Torvik C.F., Seals D.F., Randall S.K. (2002). The Calcium-Binding Activity of a Vacuole-Associated, Dehydrin-Like Protein Is Regulated by Phosphorylation. Plant Physiol..

[239] Koag M.-C., Wilkens S., Fenton R.D., Resnik J., Vo E., Close T.J. (2009). The K-Segment of Maize DHN1 Mediates Binding to Anionic Phospholipid Vesicles and Concomitant Structural Changes. Plant Physiol..

[240] Bravo L.A., Close T.J., Corcuera L.J., Guy C.L. (1999). Characterization of an 80-kDa dehydrin-like protein in barley responsive to cold acclimation. Physiol. Plant..

[241] Nylander M., Svensson J., Palva E.T., Welin B.V. (2001). Stress-induced accumulation and tissue-specific localization of dehydrins in Arabidopsis thaliana. Plant Mol. Biol..

[242] Houde M., Daniel C., Lachapelle M., Allard F., Laliberté S., Sarhan F. (1995). Immunolocalization of freezing-tolerance-associated proteins in the cytoplasm and nucleoplasm of wheat crown tissues. Plant J..

[243] Ellis R.J. (1990). Molecular Chaperones: The Plant Connection. Science.

[244] Wang W., Vinocur B., Shoseyov O., Altman A. (2004). Role of plant heat-shock proteins and molecular chaperones in the abiotic stress response. Trends Plant Sci..

[245] Gupta S.C., Sharma A., Mishra M., Mishra R.K., Chowdhuri D.K. (2010). Heat shock proteins in toxicology: How close and how far?. Life Sci..

[246] Park C.-J., Seo Y.-S. (2015). Heat Shock Proteins: A Review of the Molecular Chaperones for Plant Immunity. Plant Pathol. J..

[247] Bae M.S., Cho E.J., Choi E.-Y., Park O.K. (2003). Analysis of the Arabidopsis nuclear proteome and its response to cold stress. Plant J..

[248] Kawamura Y., Uemura M. (2003). Mass spectrometric approach for identifying putative plasma membrane proteins of Arabidopsis leaves associated with cold acclimation. Plant J..

[249] Kagale S., Divi U.K., Krochko J.E., Keller W.A., Krishna P. (2007). Brassinosteroid confers tolerance in Arabidopsis thaliana and Brassica napus to a range of abiotic stresses. Planta.

[250] Sewelam N., Kazan K., Hüdig M., Maurino V.G., Schenk P.M. (2019). The AtHSP17.4C1 Gene Expression Is Mediated by Diverse Signals that Link Biotic and Abiotic Stress Factors with ROS and Can Be a Useful Molecular Marker for Oxidative Stress. Int. J. Mol. Sci..

[251] Sasaki K., Kim M.-H., Imai R. (2007). Arabidopsis COLD SHOCK DOMAIN PROTEIN2 is a RNA chaperone that is regulated by cold and developmental signals. Biochem. Biophys. Res. Commun..

[252] Kim J.S., Jung H.J., Lee H.J., Kim K.A., Goh C.-H., Woo Y., Oh S.H., Han Y.S., Kang H. (2008). Glycine-rich RNA-binding protein7 affects abiotic stress responses by regulating stomata opening and closing in Arabidopsis thaliana. Plant J..

[253] Kim J.Y., Park S.J., Jang B., Jung C.-H., Ahn S.J., Goh C.-H., Cho K., Han O., Kang H. (2007). Functional characterization of a glycine-rich RNA-binding protein 2 in Arabidopsis thaliana under abiotic stress conditions. Plant J..

[254] Manasa S.L., Panigrahy M., Panigrahi K.C.S., Rout G.R. (2022). Overview of Cold Stress Regulation in Plants. Bot. Rev..

[255] Sengupta S., Mukherjee S., Basak P., Majumder A.L. (2015). Significance of galactinol and raffinose family oligosaccharide synthesis in plants. Front. Plant Sci..

[256] Berrocal-Lobo M., Ibanez C., Acebo P., Ramos A., Perez-Solis E., Collada C., Casado R., Aragoncillo C., Allona I. (2011). Identification of a homolog of Arabidopsis DSP4 (SEX4) in chestnut: Its induction and accumulation in stem amyloplasts during winter or in response to the cold. Plant Cell Environ..

[257] Ibáñez C., Collada C., Casado R., González-Melendi P., Aragoncillo C., Allona I. (2013). Winter induction of the galactinol synthase gene is associated with endodormancy in chestnut trees. Trees.

[258] Ko J.-H., Prassinos C., Keathley D., Han K.-H. (2011). Novel aspects of transcriptional regulation in the winter survival and maintenance mechanism of poplar. Tree Physiol..

[259] Bravo L.A., Gallardo J., Navarrete A., Olave N., Martínez J., Alberdi M., Close T.J., Corcuera L.J. (2003). Cryoprotective activity of a cold-induced dehydrin purified from barley. Physiol. Plant..

[260] Verbruggen N., Hermans C. (2008). Proline accumulation in plants: A review. Amino Acids.

[261] Puhakainen T., Hess M.W., Mäkelä P., Svensson J., Heino P., Palva E.T. (2004). Overexpression of Multiple Dehydrin Genes Enhances Tolerance to Freezing Stress in Arabidopsis. Plant Mol. Biol..

